# Production of human entorhinal stellate cell-like cells by forward programming shows an important role of Foxp1 in reprogramming

**DOI:** 10.3389/fcell.2022.976549

**Published:** 2022-08-15

**Authors:** Tobias Bergmann, Yong Liu, Jonathan Skov, Leo Mogus, Julie Lee, Ulrich Pfisterer, Louis-Francois Handfield, Andrea Asenjo-Martinez, Irene Lisa-Vargas, Stefan E. Seemann, Jimmy Tsz Hang Lee, Nikolaos Patikas, Birgitte Rahbek Kornum, Mark Denham, Poul Hyttel, Menno P. Witter, Jan Gorodkin, Tune H. Pers, Martin Hemberg, Konstantin Khodosevich, Vanessa Jane Hall

**Affiliations:** ^1^ Group of Brain Development and Disease, Department of Veterinary and Animal Sciences, Faculty of Health and Medical Sciences, University of Copenhagen, Frederiksberg, Denmark; ^2^ Novo Nordisk Foundation Center for Stem Cell Research, DanStem University of Copenhagen, Copenhagen, Denmark; ^3^ Novo Nordisk Foundation Center for Basic Metabolic Research, Faculty of Health and Medical Sciences, University of Copenhagen, Copenhagen, Denmark; ^4^ Biotech Research and Innovation Centre (BRIC), Faculty of Health and Medical Sciences, University of Copenhagen, Copenhagen, Denmark; ^5^ Wellcome Sanger Institute, Wellcome Genome Campus, Hinxton, United Kingdom; ^6^ Center for non-coding RNA in Technology and Health, Department of Veterinary and Animal Sciences, Faculty of Health and Medical Sciences, University of Copenhagen, Frederiksberg, Denmark; ^7^ Department of Neuroscience, Faculty of Health and Medical Sciences, University of Copenhagen, Copenhagen, Denmark; ^8^ Danish Research Institute of Translational Neuroscience (DANDRITE), Nordic EMBL Partnership for Molecular Medicine, Aarhus University, Aarhus, Denmark; ^9^ Disease, Stem Cells and Embryology, Department of Veterinary and Animal Sciences, Faculty of Health and Medical Sciences, University of Copenhagen, Frederiksberg, Denmark; ^10^ Kavli Institute for Systems Neuroscience, Faculty of Medicine and Health Sciences, Norwegian University of Science and Technology, Trondheim, Norway

**Keywords:** medial entorhinal cortex, forward programming, stellate cells, FOXP1, induced pluripotent stem cells

## Abstract

Stellate cells are principal neurons in the entorhinal cortex that contribute to spatial processing. They also play a role in the context of Alzheimer’s disease as they accumulate Amyloid beta early in the disease. Producing human stellate cells from pluripotent stem cells would allow researchers to study early mechanisms of Alzheimer’s disease, however, no protocols currently exist for producing such cells. In order to develop novel stem cell protocols, we characterize at high resolution the development of the porcine medial entorhinal cortex by tracing neuronal and glial subtypes from mid-gestation to the adult brain to identify the transcriptomic profile of progenitor and adult stellate cells. Importantly, we could confirm the robustness of our data by extracting developmental factors from the identified intermediate stellate cell cluster and implemented these factors to generate putative intermediate stellate cells from human induced pluripotent stem cells. Six transcription factors identified from the stellate cell cluster including *RUNX1T1*, *SOX5*, *FOXP1*, *MEF2C*, *TCF4*, *EYA2* were overexpressed using a forward programming approach to produce neurons expressing a unique combination of *RELN*, *SATB2*, *LEF1* and BCL11B observed in stellate cells. Further analyses of the individual transcription factors led to the discovery that *FOXP1* is critical in the reprogramming process and omission of *RUNX1T1* and *EYA2* enhances neuron conversion. Our findings contribute not only to the profiling of cell types within the developing and adult brain’s medial entorhinal cortex but also provides proof-of-concept for using scRNAseq data to produce entorhinal intermediate stellate cells from human pluripotent stem cells *in-vitro*.

## Introduction

The entorhinal cortex (EC) has a pertinent role in the processing of memory and navigation in the brain, which is dependent on its intrinsic organization and extrinsic connectivity (Olton et al., 1982). Stellate cells are a principal neuron subtype located in the medial entorhinal cortex (MEC) (Wigstrom, 1977) which contribute dynamically within grid circuits to process spatial memory (Rowland et al., 2018; Pastoll et al., 2020). They are a particularly interesting cell type in the context of Alzheimer’s disease (AD) since they are the first cells in the MEC to accumulate intracellular Amyloid beta both in a rat model of AD and in a small number of human patients with AD-related pathology (Kobro-Flatmoen et al., 2016). These cells are located across the cortical layers in the MEC (Sousa-Pinto, 1973), however, are the most abundant cell type found in Layer (L) II (Gatome et al., 2010). They can also be identified from pyramidal neurons by their expression of Reelin (Perez-Garcia et al., 2001). Interestingly, LII of the EC is where dramatic neuronal loss occurs, in patients with mild cognitive impairment and AD (Gomez-Isla et al., 1996; Kordower et al., 2001). Given spatial processing is also perturbed early on in AD (Henderson et al., 1989), this data suggests stellate cells are an interesting cell type to study, in respect to determining mechanisms in early AD and in understanding why stellate cells are affected prior to other surrounding cell types. Studying stellate cells derived from pluripotent stem cells could overcome the difficulties in obtaining EC tissue from healthy individuals or AD patients and allow for extensive study of this cell type *ex-vivo*. Despite obvious advantages in this approach, no protocols exist for producing stellate cells, nor any other entorhinal neurons from pluripotent stem cells. In order to produce such a protocol, local growth factors important in the development of the entorhinal cortex need to be determined for development of an indirect differentiation protocol. Alternately, specific transcription factors important for differentiation into stellate cells need to be identified if a direct programming protocol is to be developed. Advantages exist for both reprogramming approaches. For example, indirect differentiation is a non-transgenic approach that mimics normal differentiation events, whilst direct or forward programming includes a faster and more efficient conversion with less cell heterogeneity (Lujan and Wernig, 2013).

Identification of the genetic identity of stellate cell progenitors and adult stellate cells would be useful for developing a novel reprogramming protocol for pluripotent stem cells. A clearer understanding of the molecular landscape of the developing MEC will also be particularly important for disorders where perturbations in the developing EC have been identified, such as schizophrenia (Arnold et al., 1991), Sanfilippo syndrome Type B (Ohmi et al., 2011) and autism (Wegiel et al., 2010). Single-cell RNA sequencing (scRNA-seq) studies have been performed on the adult EC (Grubman et al., 2019; Franjic et al., 2021; Leng et al., 2021), but so far, no data is known on the developing EC nor on subtypes specifically found in the MEC. Neither is there a clear consensus on the transcriptomic identity of the stellate cell. Considering difficulties in obtaining human fetal tissues from the second and third trimester to investigate MEC development, we decided upon using an alternate large mammal, the pig. The pig develops a gyrencephalic brain midway through gestation, similar to humans and our recent research confirms it to be an excellent model of the developing human MEC that recapitulates neurogenesis more closely in the human than rodents (Liu et al., 2021). Genetically, the porcine genome is also more similar to human than other model organisms, with a 3-fold greater matching similarity across the genome compared to mouse-to-human sequences (Humphray et al., 2007). Together, this suggests the pig is a highly relevant model to consider in both brain development and transcriptomics.

In this study, we determine the molecular profiles of the neural cell types within the developing and postnatal porcine MEC using scRNA-seq. We focus our attention on the entorhinal progenitors and adult neurons and identify excitatory entorhinal progenitor populations and two adult excitatory neuron *RELN +* populations. We then identify potential transcriptional regulators of differentiation in one interesting *RELN* + excitatory neuron populations using a differential expression approach. We transduce six selected transcription factors using a lentiviral overexpression approach and directly forward program human induced pluripotent stem cells (iPSCs) into putative stellate cells which have relevance for modelling AD. We also identify that Foxp1 acts as a master regulator in the forward programming process.

## Materials and methods

### Animal welfare and collection of brains

The experiments conform to the relevant regulatory standards for use of fetal material. Crossbred male and female pig fetuses at Embryonic day (E) 50, E60, and E70 of development were obtained from inseminated Danish Landrace/Yorkshire sows with Duroc boar semen from a local pig production farm. Deceased postnatal pigs were obtained at postnatal day (P) 75 as a gift from Per Torp Sangild at the University of Copenhagen. Adult brains were obtained from sows killed for another study using an overdose of sodium phenobarbital by a professional issued with a license from the Danish Animal Experiment Inspectorate.

### Single-cell preparation

In total we prepared 10 sequencing-libraries form isolated MECs from E50 (whole cell, three brains, one batch), E60 (whole cell, four brains, three batches), E70 (whole cell, four brains, three batches; nucleus one brain, one batch) and from adult sow MEC (whole cell, one brain, one batch; nucleus, one brain, one batch) (Supplementary Table S1). Briefly, the individual MEC tissue was digested using a papain dissociation method, according to the manufacturer’s guidelines (Worthington) with small modifications.

The MEC was macroscopically dissected out (approx. 1 mm^3^) in the digest medium [1x PBS (Thermo Fisher Scientific), 1x Penicillin-Streptomycin (Sigma-Aldrich)], transferred to a 3.5 cm Petri dish and incubated in 1 ml papain solution for 30 min at 37°C. Dissections were performed based on our previous detailed annotations of the porcine MEC and LEC (Liu et al., 2021). The tissue was gently triturated 20 times. The cell suspension was diluted with 1 ml FBS (BioWest) and centrifuged for 5 min at 300 g at room temperature (RT). The supernatant was discarded, and the cell pellet was resuspended in 2.7 ml digestion media [1x Neurobasal medium (Thermo Fisher Scientific), 10% FBS (BioWest), 1x Penicillin-Streptomycin (Sigma-Aldrich)], 300 ul albumin-ovomucoid inhibitor, and 150 ul DNAse solution. The cell suspension was carefully layered on top of 5 ml of albumin-inhibitor solution in a 15 ml falcon tube and centrifuged for 6 min at 70 g at RT. The supernatant was discarded, and the cell pellet was resuspended in 5 ml cell-resuspension media (1x Neurobasal medium (Thermo Fisher Scientific), B27 (Thermo Fisher Scientific), 1x Penicillin-Streptomycin (Sigma-Aldrich), bFGF (5 ng/ml, Prospec). The cells were counted (NucleoCounter, ChemoMetec) and ranged in viability from 80.4–88.4% (Viability and Cell Count assay), diluted to 100–2000 cells/ul used for single-cell library preparation.

### Single-nuclei isolation

Nuclei extraction was performed as described before (Krishnaswami et al., 2016) with the following modifications (Pfisterer et al., 2020). Prior to nuclei extraction, nuclei isolation medium 1 (NIM1) (250 mM sucrose, 25 mM KCl, 5 mM MgCl2, 10 mM Tris Buffer pH8), NIM2 (NIM1 buffer supplemented with 1 μM DTT (Thermo Fisher Scientific) and 1x EDTA-free protease inhibitors (Roche) and homogenization [NIM2 buffer supplemented with Recombinant RNase Inhibitor (0.4 U/μl, Takara), SUPERase in (0.2 U/μl, Thermo Fisher Scientific) and Triton (0.1% v/v, Sigma-Aldrich)] buffers were prepared. Briefly, sectioned frozen brain tissue was placed into pre-cooled 1 ml dounce homogenizer (Wheaton) with 1 ml ice-cooled homogenization buffer. Tissue was dissociated on ice using 5-6 strokes with the loose pestle and 15–17 strokes with the tight pestle. Homogenate was first filtered through a 70 μm filter. Nuclei were collected (900 g, 10 min) and resuspended in 500 μl staining buffer [nuclease free PBS (1X, Thermo Fisher Scientific), BSA (0.5% wt/vol, Sigma-Aldrich), SUPERase in (0.2 U/μl, Thermo Fisher Scientific)]. The sample was stained with 7-AAD (2 ug/ul, Sigma-Aldrich) in order to visualize nuclei during FACS sorting. 7-AAD positive nuclei were FACS-isolated (70 μm nozzle, BD Biosciences, BD FACSAria™) into a 1.5 ml Eppendorf tube containing 10 μl 10% nuclease free BSA (Thermo Fisher Scientific). Single 7-AAD + nuclei were isolated using the gating strategy like Pfisterer et al. (2020).

### Single-nuclei RNA-seq library preparation and sequencing

Whole cells were loaded onto the 10X Genomics microfluidic chip according to the Chromium Single Cell 3′ Reagent Kits User Guide version 2 chemistry (10X Genomics). The single-nuclei samples were loaded as per whole cells, albeit the samples were not diluted. 10–12.000 thousand cells/nuclei were loaded from each sample.

Libraries from two samples at different stages were pooled and sequenced together on an Illumina NextSeq 500 (Supplementary Table S1) following the NextSeq System Denature and Dilute Libraries Guide Protocol A: Standard Normalization Method (illumina). The NextSeq 500/550 High Output Reagent Cartridge v2 75 cycles (illumina) kit was used for the whole cell and single-nuclei samples and the pooled library were sequenced on NextSeq 500. The sequencing cycles were: Read1, 26 cycles, i7 Index eight cycles, i5 Index 0 cycles; Read2, 57 cycles.

### Plasmid design and construction

The selected genes of interest (GOIs) were cloned into the pTet-O-Ngn2-puro plasmid (Addgene #52047) by replacing *Ngn2* with the GOI. This ensured that the GOI was fused to the puromycin resistance gene. Plasmids containing *EYA2* (Addgene #49264), RUNX1T1 (Addgene #49264), *TCF4* (Addgene #109144), *Foxp1* (Addgene #16362), *Sox5* (Addgene #48707) and *MEF2C* (Addgene #61538) were all acquired from Addgene. Forward and reverse primers were designed for subcloning all GOIs (except for MEF2C which was already subcloned into a lentiviral Tet-O plasmid (Addgene #61538) (see Supplementary Table S2 for primer sequences). Gene inserts were amplified from the plasmids by PCR amplification using 400–600 ng template plasmid, 0.5 µM forward and reverse primer (Eurofins Genomics), 5 µl Pfu DNA Polymerase 10X Buffer (Promega), 1 µl Pfu DNA Polymerase (Promega), and 200 µM dNTP mix (Promega) in a 50 µl reaction volume. The following program was used in a PTC-200 Thermal Cycler (MJ Research): one cycle of 2 min at 95°C, 40 cycles of 1 min at 95°C, 30 s at 60°C, and 1 min at 72°C. The PCR amplified inserts were run on a 1% agarose gel and purified using the Wizard SV Gel and PCR Clean-Up System kit (Promega) according to the manufacturer’s instructions. The gene insert was then ligated into a Zero Blunt TOPO vector using the Zero Blunt TOPO PCR Cloning Kit (Invitrogen) according to the manufacturer’s protocol. The TOPO vector containing the gene insert was heat-shock transformed into competent *E. coli* (NEB Stable C3040I) and grown at 37°C O/N on agar (Sigma-Aldrich) plates containing 50 mg/ml kanamycin (Sigma-Aldrich) for selection. Individual colonies were propagated at 37°C O/N in 5 ml Luria-Bertani (LB) broth (Sigma-Aldrich) supplemented with 50 mg/ml kanamycin. Plasmids were isolated using the PureYield Plasmid Miniprep System (Promega) according to the manufacturer’s protocol. The GOIs were isolated from the purified plasmids by digestion with EcoRI (Esp3I for RUNX1T1) and XbaI according to the manufacturer’s protocol (FastDigest, Thermo Fisher Scientific). Positive clones were purified from a 1% agarose gel. The GOI was then inserted and ligated into a dephosphorylated pTet-O-Ngn2-puro (Addgene 52047) backbone to create the final plasmid; pTet-O-GOI-puro using the LigaFast Rapid DNA Ligation System (Promega) and heat-shock transformed into competent *E. coli*. Individual colonies were selected and isolated using the PureYield Plasmid Miniprep System (Promega) according to the manufacturer’s protocol. Correct plasmid inserts were validated by digestion with EcoRI and XbaI (FastDigest, Thermo Scientific). The plasmids were verified by Sanger sequencing (Eurofins Genomics) and stored at −80°C until use for virus production.

### Lentiviral production and titering

Second generation lentiviruses were produced following transfection of HEK293FT cells. On the day of transfection, HEK293FT cells were cultured in fresh DMEM supplemented with 10% newborn bovine calf serum (NCS) (Hyclone, New Zealand) in the absence of antibiotics. The cells were transfected as follows: In one tube, 50 µl Lipofectamine 3000 reagent (Invitrogen) was diluted in 1.5 ml Opti-MEM (Gibco; Thermo Fisher Scientific). 40 µl P3000 reagent (Invitrogen) and 5 pmol DNA plasmids (1.5 pmol psPAX2 (Addgene #12260), 1.5 pmol pMD2.G (Addgene #12259) and 2 pmol transfer plasmid containing the GOI) were diluted in 1.5 ml Opti-MEM. The two tubes were mixed and incubated at RT for 10 min and the solution was then added dropwise to the cells. After 24 h the media was replaced with fresh DMEM supplemented with 10% NCS and incubated for 48 h. The virus was harvested 72 h post transfection. The virus was spun at 500 g for 5 min and the supernatant filtered through a 45 µm polyether sulfone filter (Fisher Scientific) and subsequently concentrated, by spinning at 23,000 rpm (using rotor Beckman Type 60 Ti) for 2 h at 4°C. The supernatant was discarded and 100 µl PBS was added to the pellet and incubated for 1 h at 4°C. The pellet was resuspended by pipetting and stored at −80°C until use for transduction.

Titering of virus was performed on HEK293FT cells by adding lentivirus at different amounts (from 1 to 15 µl virus). A virus with a predetermined titer was added as a reference and a negative control without virus was included. On Day 1, the media was substituted with fresh media, and 48 h (Day 2) after transduction, the genomic DNA (gDNA) was extracted from the cells using the PureLink Genomic DNA Mini Kit (Invitrogen) according to the manufacturer’s protocol. Each sample was analyzed in triplicate by qPCR using both primers for a sequence fused to the GOI (LV2), fused to all GOIs, and for the β-actin intron (see Supplementary Table S3 for primer sequences). The LV2 was used to determine the degree of viral integration into the genome. The β-actin gene was used to determine the number of genome copies, as an estimate for the total number of cells. For the qPCR reaction, 5 µl SYBR green (Roche), 1 µl 10 mM of both forward and reverse primer and 15 ng gDNA was mixed in a 10 µl reaction volume and loaded in a white qPCR plate with optical caps (Thermo Fisher Scientific). Samples were spun briefly and run on a Mx3005P qPCR machine (Agilent) using the following program: one cycle of 10 min at 95°C, 40 cycles of 20 s at 95°C, 20 s at 55°C, and 30 s at 72°C, followed by a melting curve analysis.

The viral titers were determined based on a recently published approach with minor variations (Gill and Denham, 2020). Briefly Ct values were converted from their intrinsic exponential relation to linear related Xo values, using the Xo method (Thomsen et al., 2010). The triplicate LV2 Xo mean values for a sample was normalized to its mean Xo value for β-actin to obtain the copy number of integrated GOI relative to β-actin gene copies in the given sample. A standard curve of normalized LV2 sequence copy number versus the volume of virus that was added to the sample initially was fitted for each titration of the virus, respectively. All curves followed a linear relationship with R2 ranging from 0.889 to 0.995. The relative GOI integration for the reference virus was then calculated and inserted into the linear regression curves for each respective virus, where (VPD, volume of produced virus): VPD = a * relative copy number of reference virus + b. The VPD is a measure of the volume of the produced virus that is needed, under the same conditions as the reference virus, to obtain the same degree of GOI integration as was obtained for the reference virus. The VPD for each of the produced viruses was hence calculated by inserting the relative copy number of the reference virus in the linear regression for the respective virus produced. The titer of the produced virus was finally calculated based on the titer of the reference virus, using a multiplicity of infection (MOI) of 10, where U is units of viral particles: VPD = (Number of cells * MOI)/Titer (U/µl).

### Cell culture

For direct reprogramming, three human iPSC lines were selected including the human SFC180-01-01/StBCi064-A (female donor, healthy, age unknown) (acquired from the EBiSC cell bank) and SBAD-03-01 (female donor, healthy, age 32) and SBAD-02-01 (male donor, healthy, age 51) (acquired from Oxford Uni donated by Zameel Cader’s lab). The cells were maintained in Geltrex (Gibco; Thermo Fisher Scientific) coated dishes with mTESR media (Stem Cell Technologies) supplemented with 1% Penicillin-Streptomycin (Gibco; Thermo Fisher Scientific). The cells were passaged using 0.5 mM EDTA (Merck) and propagated until they reached 50–60% confluency, before initiating reprogramming using the produced lentivirus. The cell lines were cultured at 37°C in 5% CO2 with 90% humidity.

### Transduction of human induced pluripotent stem cells

Human iPSCs were transduced in mTESR in the absence of antibiotics. The lentiviruses containing the GOIs were transduced using five MOI in the presence of the reverse tetracycline transactivator M2rtTA (MOI 5). After 3 h the media was supplemented with half the usual volume of fresh media. The day after viral transduction was considered Day 0, and the media was exchanged for medial pallium (MP) patterning media [50:50 Neurobasal A medium: DMEM/F12 containing Glutamax (Thermo Fisher Scientific), 1% Insulin-Transferrin-Selenium A (Gibco; Thermo Fisher Scientific), 2% B-27 Supplement minus Vit A (Gibco; Thermo Fisher Scientific), 1% N-2 supplement (Gibco; Thermo Fisher Scientific), 0.3% Glucose, 3 µM CHIR 99021 (Sigma-Aldrich), 5 nM BMP4 (R&D systems), 1 μg/ml doxycycline (Sigma-Aldrich)]. On Day 2 and 3 the media was exchanged for MP patterning media supplemented with 3 μg/ml puromycin to select for transduced cells. On Day 5, the cells were dissociated using Accutase (Corning), spun for 5 min at 300 g and cultured in NBM media (96% Neurobasal A medium, 2% B-27 Supplement, 1% GlutaMAX, 1% pen strep, 1 μg/ml doxycycline, 1 µM DAPT (Sigma-Aldrich) and 3 μg/ml puromycin). Upon confluency, the cells were split onto triple coated (1x poly-L-ornithine (Sigma) 10 μg/ml fibronectin (Sigma-Aldrich), 10 μg/ml Poly-D laminin (Sigma-Aldrich)) plates and medium was exchanged every second day. The cells were propagated in the NBM medium without puromycin from D7 onwards and up to D30.

### Immunocytochemistry

The differentiated human iPSCs were fixed on glass coverslips (VWR, Denmark) in 4% PFA for 15 min at RT and stored at 4°C until use. The cells were washed in PBS and permeabilized in 0.1% Triton X-100 in PBS for 1 h, washed in PBS and antigen retrieval was performed by immersing cells into boiling one x citrate buffer 3 times for 5 min each. Cells were treated with blocking buffer (5% NDS in PBS) for 1 h at RT. Cells were incubated overnight at four°C in primary antibodies targeted against BCL11B (1:750, Abcam, ab18465), Reelin (1:50, Santa-Cruz, sc-25346), SATB2 (1:800, Abcam ab34735), Nestin (1:500, Millipore, Abd69) and MAP2ab (1:200, Sigma, M1406) diluted in blocking buffer. The cells were washed 3 × 5 min in PBS and incubated with secondary antibodies conjugated with Alexa fluorophores (1:200, Invitrogen, A10036, A21208, and A21448) diluted in blocking buffer for 1 h at RT. The cells were then washed three times for 5 min in PBS and counterstained with Hoechst 33342 (1 μg/ml in PBS) for 10 min. The cells were washed in PBS and mounted onto glass microscope slides using buffered glycerol mounting media 90% glycerol (Sigma-Aldrich), 20 mM Tris pH 8.0, 0.5% N-propyl gallate (Sigma-Aldrich).

### Image processing and quantification

For acquisition of immunocytochemistry images, a confocal microscope Leica TCS SPE was used. Quantification of immunostainings was performed using the ImageJ 1.53k. Automated cell counting was performed using the Analyze particles tool after converting images to greyscale and using the threshold and watershed tools to highlight the cells to be analyzed.

### qPCR

Total RNA was extracted from cells using TRIzol reagent (Life Technologies) according to the manufacturer’s protocol. After isolation of the aqueous phase 10 µg of glycogen (Thermo Fisher Scientific) was supplemented before precipitation of the RNA. The pellet was resuspended in nuclease free water and the concentration was determined using a NanoDrop. Synthesis of cDNA was performed using the High-Capacity cDNA RT kit (Applied Biosystems) with random hexamer primers, according to the manufacturer’s protocol. The qPCR was performed in biological triplicates. For the qPCR reaction, 5 µL SYBR green (Applied Biosystems), 1 µl 10 mM of both forward and reverse primer (see Supplementary Table S3 for primer sequences) and 25 ng cDNA was mixed in a 10 µl reaction volume and loaded in a qPCR plate with optical caps (Thermo Fisher Scientific). Samples were spun down briefly and run on a Mx3005P qPCR machine (Agilent) using the following program: one cycle of 10 min at 95°C, 40 cycles of 20 s at 95°C, 20 s at 55°C, and 30 s at 72°C, followed by a melting curve analysis. Statistics and graphs were performed in Excel and Prism 8 (GraphPad Software). Gene expression was calculated using the 2^−△△Ct^ method. CT values were normalized to the housekeeping gene, *ACTIN* and △Ct was normalized to one of the three iPSC lines transduced with the lentiviruses at either D10 or D30 to form △△Ct. The SD was calculated for the individual gene investigated. A two-tailed T-test of unequal variance was performed to compare the expression between two samples to determine statistical significance. Thresholds for significance were placed at *p ≤ 0.05, **p ≤ 0.01, ***p ≤ 0.001.

### Initial quality control and data analysis

Briefly, the sequenced libraries were mapped to pre-mRNA and filtered using 10X Genomics Cell Ranger pipeline. The sequencing data was demultiplexed by bcl2fast (Illumina; version 2.2.0) which is warped in the Cell Ranger (version 2.2.0). The reads were mapped to the porcine reference genome assembly (Sscrofa11.1 release-94) using STAR (version 2.5.1b) (Dobin et al., 2013). This porcine ENSEMBL gene annotation (Warr et al., 2020) was used for all analyses. The mRNA molecules and quality of the sequencing libraries was counted and assessed by Cell Ranger using default settings, which determined in each sample the sequencing depth cutoff that is required for cells to be included in the downstream analysis. All samples were merged for downstream clustering and data analysis, which was performed in Seurat (version 2.3.4) (Satija et al., 2015). Similar numbers of UMIs and genes were observed across all cell types and batches (Supplementary Figures S7A–C). Overview of number of cells, mean reads per cell, median genes per cell, and number of reads and pooled brains in the included scRNA-seq libraries can be found in Supplementary Table S1.

### Batch correction

We used decomposeVar from the “scran” R package (version 1.8.2) in order to find a list of variable genes that are used for PCA dimensionality reduction; however, to aid the correspondence of single-cell to single-nucleus for down-stream analysis (such as clustering), we identified and excluded genes whose transcripts were highly abundant in empty droplets Empty droplets were defined as cells with <50 UMIs. If the number transcripts that are found in the empty droplet is above 30% the total number of transcripts for a given gene (Supplementary Figure S7D), that gene will be removed from the list of variable genes.

**FIGURE 1:**
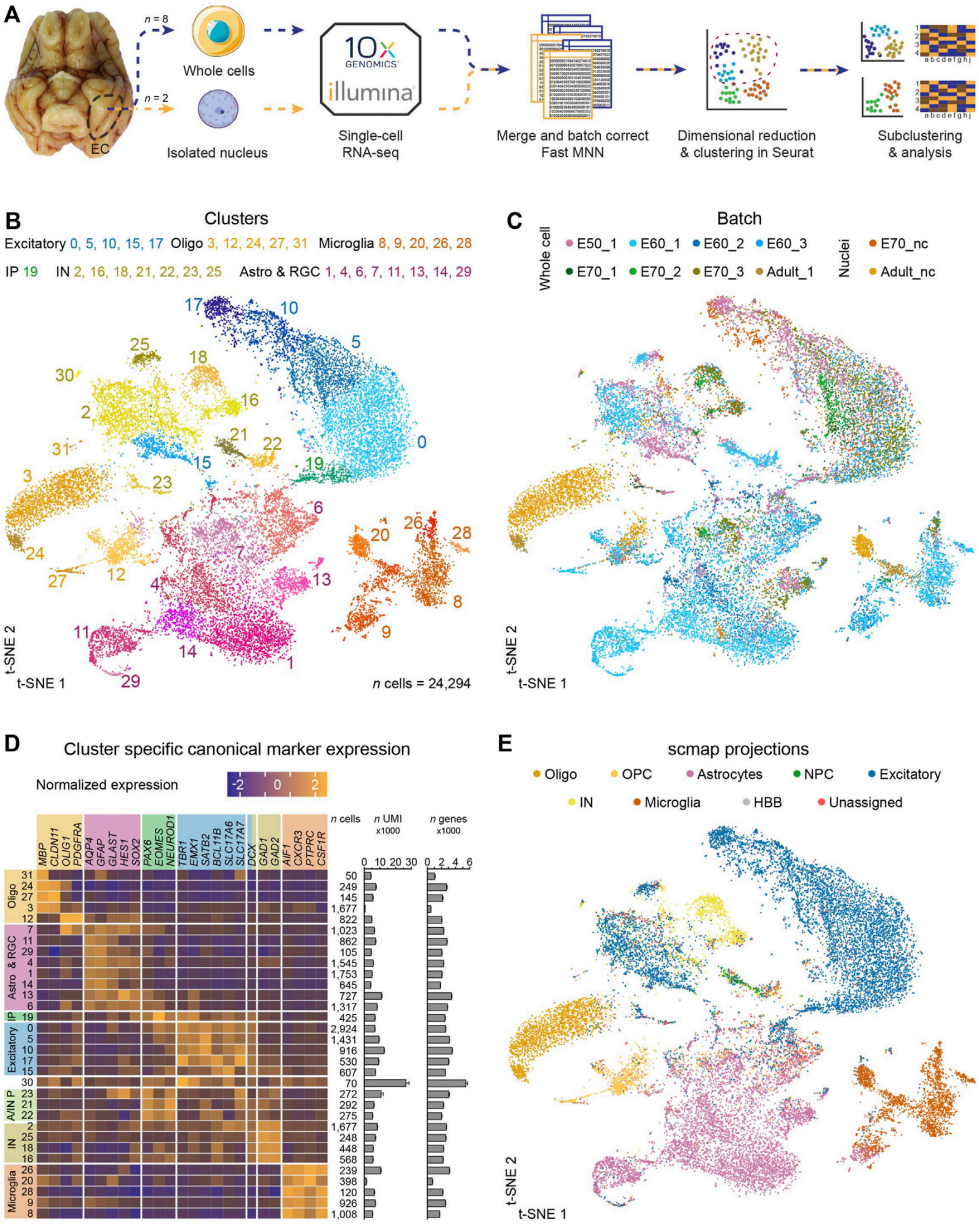
Single-cell profiling of the entorhinal cortex reveals thirty-two distinct cell clusters of major cell type populations. **(A)** The computational pipeline includes ten batches of single-cell and single-nuclei isolated cells from E50, E60, E70 and P75 which were captured using the 10X genomics platform. Batches were merged using the Fast MNN approach and dimensional reduction and clustering were performed in Seurat followed by subclustering and analyses of selected clusters. **(B)** A t-SNE plot of 24,294 cells merged from all timepoints revealed thirty-two distinct populations following FastMNN batch correction. Oligodendro-glia/-cytes (Oligo), astrocytes and radial glia (Astro & RGC), intermediate progenitors (IP), excitatory neurons (Excitatory), astrocyte and interneuron progenitors (A/IN P) and microglia (Microglia) **(C)** t-SNE plot annotated by batch demonstrates good integration of subtypes within same ages following correction by FastMNN of the 10 datasets (pre-batch correction shown in Supplementary Figure S7E). **(D)** Analysis of canonical marker genes resulted in categorization of several Oligo, Astro & RGC, IP, Excitatory, A/IN P and Microglia clusters. The number (*n*) of cells, mean UMI counts (unique transcripts) and mean number of different expressed genes for each cluster are also represented. Error bars denote the SD. **(E)** Projection of the dataset onto an already-annotated human fetal prefrontal cortex and human medial temporal gyrus single-cell dataset consolidates marker gene-driven annotation of the dataset. Abbreviations: NPC, neural progenitor cells; HBB, marker expressed in human blood cells.

The individual datasets were log-normalized and the PCA projected gene expression in the datasets were then batch corrected by FastMNN in the scran package using default parameters. (Haghverdi et al., 2018). This reduced the distance between cell pairs that are found to be reciprocally nearest neighbors across batches (before batch correction; Supplementary Figure S7E, after correction; Figure 1C). The merged dataset were visualized by t-distributed stochastic neighbour embedding (t-SNE) (Van Der Maaten and Hinton, 2008) embedded in function runTSNE in the scran package (using the dimensional reduction created by FastMNN as input and otherwise default parameters).

### Unsupervised clustering

Cell types were identified using the Louvain algorithm (Blondel et al., 2008) used in the Seurat (version 2.3.4) function FindClusters with a resolution parameter set at 1.6 for the entire dataset, 1.4 for the IN analysis, 1.2 for the oligodendrocyte analysis, and 1.0 for the IP and excitatory neuron analysis (dims.use = 1:50 and otherwise default parameters used). The canonical markers were used to identify the neurons of the clusters (Figure 1D).

### Projection of data to published datasets

To ascertain the quality of our annotated dataset, we projected the entire dataset to two reference datasets which each contained smart-seq2 single-cells from human embryonic prefrontal cortex (Zhong et al., 2018) or from human adult middle temporal gyrus (Hodge et al., 2019). We used scmap (version v1.12.0) (Kiselev et al., 2018) to project individual cells onto curated cell-type clusters that are available in each reference. The projection was based on the most 2000 and 2500 variable genes found within each reference dataset, respectively. Each cell-type prediction utilizes the consensus of three similarity measures from queried cell to reference cluster centroids using sets of cell-type markers that were identified in the respective reference datasets; however, only the human genes that possess an ortholog gene in the pig were used as cell type marker for similarity measure calculation. Scmap parameters were set to report a match in the reference with lower than the default 0.7 similarity by setting the threshold parameter to 0. Next, we wanted to assess the uniqueness of the excitatory neurons from the MEC. We did so by comparing our scRNA-seq data to droplet-based 10X Genomics scRNA-seq of temporal cortex samples from 10 non-epileptic subjects (Pfisterer et al., 2020). This study reported eight major classes of principal cells using family-layer-specific marker genes at low resolution and 31 subtypes at higher resolution. We filtered our data for excitatory neurons, log2-normalized the expression values, and merged all samples. We applied scmap (Kiselev et al., 2018) using default settings for a more stringent analysis to select features specific to both the 8 and 31 annotated cell types in the merged dataset and for each of the 10 samples separately. Finally, we projected the log2-normalized expression values of our pig scRNA-seq data containing 8,833 excitatory MEC neurons onto the cell types in human temporal cortex.

### Differential gene analysis

Marker genes were identified using scfind (version 3.7.0) (Lee et al., 2021) based on highest F1 score for cluster specific genes. In principle by: For cluster c_j_, gene gi is considered a true positive (TP) if it is expressed (defined as at least one UMI count), a false negative (FN) if it is not expressed, a false positive (FP) if is expressed in a cell assigned to another cluster, and a true negative (TN) if it is not expressed in a cell assigned to a different cluster. Gene expression is defined when at least one UMI count is detected for the gene. For each gi we evaluate precision = TP/(TP + FP), recall = TP/(TP + FN), and F1 = 2*precision*recall/(precision + recall). For each cj, genes are ranked by F1 with the highest scoring genes used as markers.

### Identification of cell type specific transcription factors

Four excitatory neuron clusters expressing *RELN*, determined as potential stellate cell populations, were investigated for cluster-specific enriched TFs. Porcine genes [identified by the reference genome (Sscrofa11.1 release-94)] were defined as TFs (total of 2475 genes) if their gene symbols perfectly matched TF gene symbols in TFcheckpoint (http://tfcheckpoint.org: downloaded 2018.05.15). The repository contains 3479 specific DNA-binding RNA polymerase II TFs from human, mouse and rat and is curated from several TF sources (Chawla et al., 2013). Differentially expressed TFs between each *RELN* cluster and all remaining cells were identified using FindMarkers in the Seurat (version 2.3.4) pipeline (Butler et al., 2018) using the Wilcoxon Rank Sum test [min.pct set to 0.25 (only testing genes expressed in at least 25% of the cells) and otherwise default parameters]. For each of the 2 *RELN* clusters, 20 TFs were selected with the highest log fold-change of average expression between the cluster and the remaining cells.

### Cell cycle score analysis

Cell cycle phases were scored using the built-in function in Seurat CellCycleScoring, which was used to determine whether a given cell was likely to be in either S, G2M or G1 (which is indistinguishable from G0) phase of cell cycle. Cell cycle scores were based on a list of cell cycle phase-specific genes proposed by Tirosh et al. (2016).

## Results

### Single-cell RNA sequencing reveals thirty-two cell populations in the developing medial entorhinal cortex

ScRNA-seq is a powerful approach for revealing the molecular identity of cell types in the different types of tissue, including the brain. We used this methodology to investigate the timing in the emergence of different cell types of the MEC and their molecular identity during development and postnatal maturation. ScRNA-seq was performed using the 10X Genomics microfluidics-based Chromium platform on whole-cells isolated from the MEC at embryonal day 50 (E50), E60, E70 and postnatal day 75 (P75), and isolated nuclei in the later stages of development at E70 and P75 to ensure efficient capture of the neurons in the tissue (Figure 1A). We chose to integrate datasets for all age groups simultaneously as opposed to analyzing time points separately to increase power and to allow us to easily cross-label cell types that existed both during fetal development and in the postnatal brain. We excluded red blood cells (expressing hemoglobin subunits *HBB*, *HBE1*, *HBM*, *HBZ* and on average, only 291 genes per cell) and vascular cells (*PDGFRB* ^*+*^ */PECAM1* ^+^) from our dataset, which left 24,294 cells (mean of 2798 expressed genes per cell) for further analyses. We log-normalized each sample dataset, merged all samples, batch corrected by FastMNN Haghverdi et al. (2018) and performed unsupervised clustering in Seurat (Figure 1A). Clustering of the merged dataset revealed thirty-two distinct cell clusters (Figure 1B) with many clusters represented by cells from datasets across different batches, showing good integration of batches within the same age (Figure 1C). The clusters could be delineated into six main cell populations demarcated by canonical markers for intermediate progenitor cells (IPs, *PAX6* ^*+*^/*EOMES* ^*+*^/*NEUROD1* ^*+*^), excitatory neurons (Excitatory, *TBR1* ^*+*^/*EMX1* ^*+*^/*SATB2* ^*+*^/*BCL11B* ^*+*^/*SLC17A6* ^*+*^/*SLC17A7* ^*+*^/*DCX* ^*+*^), GABAergic interneurons (IN, *GAD1* ^*+*^/*GAD2* ^*+*^/*DCX* ^*+*^), microglia (Microglia, *AIF1* ^*+*^/*CXCR3* ^*+*^/*PTPRC* ^*+*^/*CSF1R* ^*+*^), oligodendroglia (Oligo, *MBP* ^*+*^/*CLDN11* ^*+*^/*OLIG1* ^*+*^/*PDGFRA* ^*+*^), astrocytes (Astro, *AQP4* ^*+*^ */GFAP* ^*+*^ */GLAST* ^*+*^) and radial glia (RGC, *HES1* ^*+*^ */SOX2* ^*+*^) and astrocyte and interneuron progenitor cells (A/IN P) which had a mix of both IP markers and astrocyte or interneuron markers) (Figure 1D). Cluster 30 consists of a small number of cells (*n* = 70) which contained twice as many transcripts and genes expressed (Figure 1C) which we believed to be doublets which had not been removed by the data-preprocessing. Cluster 31 expressed *MBP* (Figure 1C), but was also omitted from the sub-analysis, as the highest enriched genes in this cluster were mitochondrial genes (Supplementary Figure S1A) and may likely be dying cells. Of the total dataset, 42% of the captured cells were neurons [GABAergic interneurons, 14% (*n* = 3,498); excitatory neurons, 28% (*n* = 6,833)], 11% were oligodendrocytes/oligodendroglia (*n* = 2,696) and the remaining 46% of cells (*n* = 11,267) were astrocytes, radial glia, intermediate progenitors and microglia. Furthermore, we validated our marker-expression driven annotations by projecting our dataset using scmap (Kiselev et al., 2018) onto a publicly available and annotated datasets of the human embryonic prefrontal cortex (Zhong et al., 2018) and the human adult middle temporal gyrus (Hodge et al., 2019) (Figure 1E). We found a high concordance between our annotation of major cell types and the human brain datasets, despite the obvious regional differences. To ascertain how unique the excitatory neuron subtypes of the MEC are compared to other neocortical areas, we projected 6,833 cells (selected from the excitatory MEC neuron clusters) to individual temporal cortex samples from 10 healthy human brains at different clustering resolutions (8 or 31 clusters) (Pfisterer et al., 2020) (Supplementary Figure S2). The 6,833 excitatory MEC neurons showed no significant similarities to the temporal cortex excitatory neurons. At the higher clustering resolution, only 25 neurons (0.3% of the total MEC excitatory neurons) from late developmental stages (E70 and adult) projected to LV/VI Fezf2_Tle4 (a cell type marking LV/VI cells in the neocortex expressing FEZF2) and to two other glutamatergic principal neuron subtypes from LIII/IV of the neocortex, LIII Cux2_Prss12 and LIV Rorb_Mme 8 (Supplementary Figure S2). This suggests the entorhinal dataset contains principally, unique excitatory neurons with very little overlap with excitatory neurons from the temporal cortex, except for only a few neurons sharing transcriptional profiles to three principal neuron subtypes.

### Progenitor and adult interneurons from both the medial ganglionic eminence and caudal ganglionic eminence are identified in the developing medial entorhinal cortex

**FIGURE 2:**
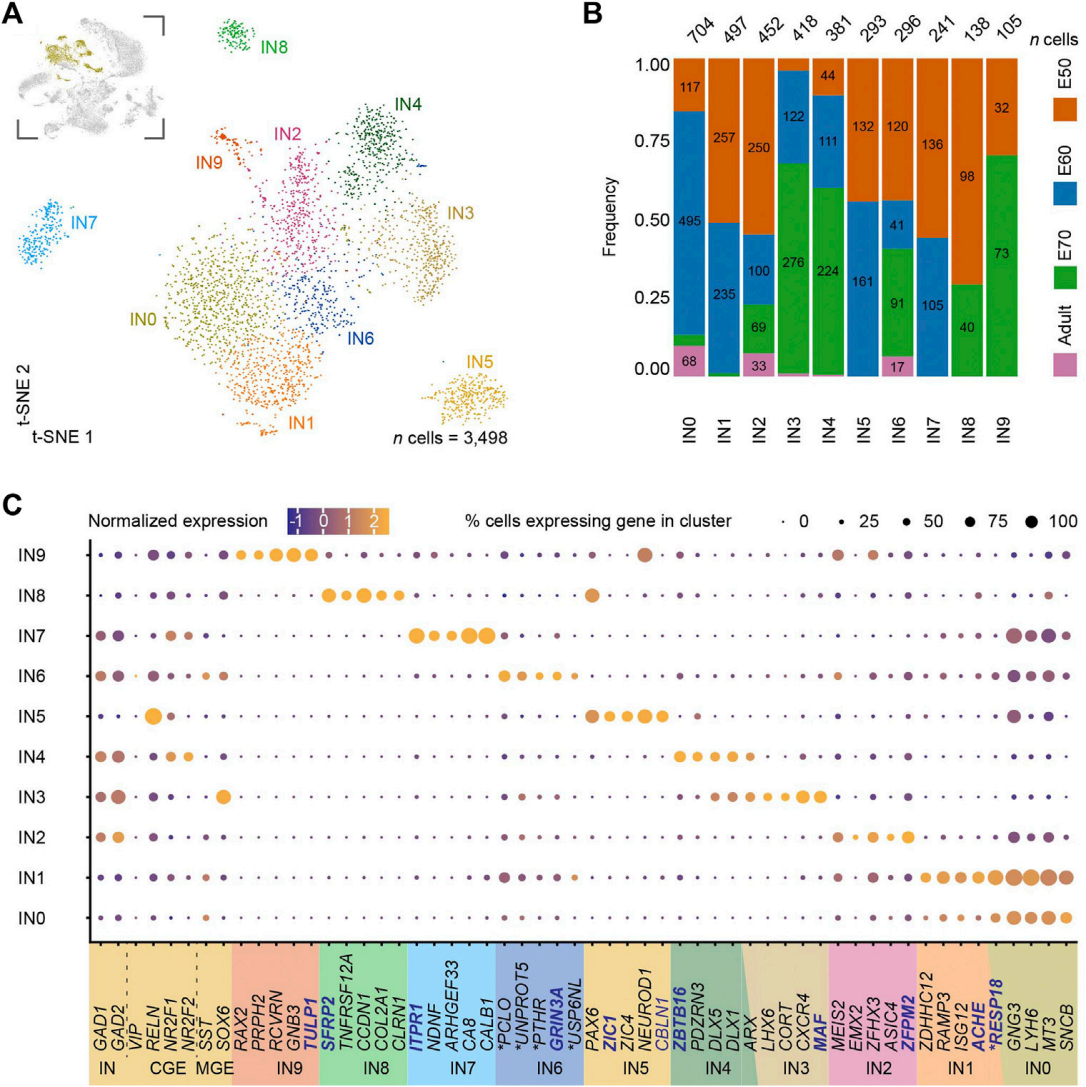
Subclustering of medial entorhinal cortex (MEC) interneurons (INs) reveals ten clusters with unique gene signatures. **(A)** A t-SNE plot of the subclustering identifying ten interneuron (IN) clusters with one cluster more transcriptionally distinct than the others. IN clusters highlighted in yellow in the inset image were subclustered from the parent dataset and are depicted in the top left corner. **(B)** An analysis of the developmental stages across the ten clusters highlights IN1, IN5, IN7, IN8 and IN9 are found exclusively during development and suggestive of IN progenitors and the remaining clusters are adult INs. **(C)** Unique gene signatures were identified for the ten clusters by differential gene expression analysis, together with the population specific expression of canonical markers for interneurons (IN) derived both from the caudal ganglionic eminence (CGE) and the medial ganglionic eminence (MGE). Localization of genes with names in blue are shown in **(D)**. The ∗ marks the human ortholog names (Supplementary Table S4).

We characterized the IN populations in the population to differentiate between the progenitor and adult cell populations in the MEC. We sub-clustered a total of 3,498 cells from the dataset using the canonical IN markers, *GAD1* and *GAD2* (clusters 2, 16, 18, 21, 22, 23 and 25) which produced ten subclusters IN0-IN9 (Figure 2A). We included clusters 2 and 25 in our IN analyses even though the scmap projection annotated these two clusters as excitatory neurons (Figures 1D,E). Despite this discrepancy, cluster 25 expressed IN genes, such as *GAD1* and *GAD2* and lacked *SLC17A6/7* and became IN7 after subclustering. For cluster 2 from the main dataset (mainly present at E60), a closer analysis of canonical markers suggested an ambiguous identity as the cells expressed both the excitatory markers, *SLC17A6/7* and IN markers, *GAD1/2* (Figures 1B,D). The unique molecular identifier (UMI) counts for this cluster were normal, therefore, it is unlikely to contain doublets. Cluster 2 when subclustered spread across the IN0-IN2 and IN6 populations. Five of the IN populations were only detected during development, suggesting they may be immature INs (IN1, IN5, IN7, IN8 and IN9). Three of these, IN5, IN8 and IN9 express *PAX6* further confirming this finding. IN5, IN7 and IN8 also clustered further away from the other IN clusters which also suggests they are more transcriptionally distinct than the other IN clusters (Figure 2A). The remaining IN populations, IN0, IN2, IN3, IN4 and IN6 were all found in the postnatal brain and are likely mature IN populations (Figure 2B). We could identify several well-known IN markers that were either expressed broadly or label specific IN cell subtypes, including, *CALB1*, *DLX1*, *DLX5*, *GAD1*, *GAD2*, *LHX6*, *NPY*, *RELN*, *SST*, and *SOX6*, (Figure 2C). However, we were unable to identify other IN markers, *HTR3A*, *PVALB*, *CCK*, *NOS1*, *LHX5* and *VIP*, likely due to low expression levels and low sequencing depth*.* Based on *SST* expression in IN0, IN2 and *LHX6* and *SOX6* in IN3, we assign these clusters as MGE-derived INs (Figure 2C). CGE-derived INs were identified based on expression of Coup TF1 (*NR2F1* gene), CoupTF2 (*NR2F2* gene) (Kanatani et al., 2008) and *NDNF* (Tasic et al., 2018) or in the case of IN4, *NR2F1* and *NR2F2* (Figure 2C). The remaining immature populations IN5, IN7-IN9 clusters also expressed these CGE IN markers (Figure 2C). Together we identify five immature and five mature IN transcriptomic profiles in the developing EC from both CGE and MGE origins.

### Single-cell RNA sequencing analysis reveals that oligodendrocyte progenitor cells emerge in the early medial entorhinal cortex

We then assessed the oligodendroglia populations within the MEC, which consisted of oligodendrocyte progenitor cells (OPCs) and oligodendrocytes at various stages of maturation. Subclustering and subsequent analyses were performed on clusters 3, 12, 24, 27 (Supplementary Figure S3A insert). Five distinct clusters were identified from the subclustering (Supplementary Figures S3A–C). Two clusters (OPC0 and 1) expressed OPC markers *PDGFRA* and *OLIG1* (Supplementary Figure S3C), with a large proportion of the cells undergoing cell cycle division (50 and 25% respectively, Supplementary Figure S3D). We were unable to identify *OLIG2* in these two clusters, likely due to low expression levels and sequencing depth. However, this data suggested these two clusters were progenitor cells. Cluster OPC1 was composed of mostly fetal cells from E50 to E70 whilst cluster OPC0 was detected both during gestation and postnatally (Supplementary Figure S3B). OPC0 and OPC1 are clearly two separate populations, as both populations are detected after birth, albeit the clustering suggests a larger population of OPC0 after birth. Several genes have been previously identified in OPCs. For example, cluster OPC0 expressed the genes *LHFPL3* and *MMP16*, and cluster OPC1, *STMN1*; previously identified in rodent OPCs (Lin et al., 2009; Hu et al., 2011; Magri et al., 2014; Artegiani et al., 2017). Interestingly, our data revealed an additional population, OLI2, that was constituted predominantly of fetal cells from E60 OLI2 (Supplementary Figures S3A–C). These cells surprisingly also expressed the mature oligodendrocyte markers, *MBP*, *CLDN11*, *GRP17*, *NKX2-2*, and *MAG* (Supplementary Figure S3C). Together, this data suggests that a small population of myelinating oligodendrocytes might be present in the fetal porcine MEC already at E60. The oligodendrocyte clusters OLI3 and OLI4 consisted almost exclusively of adult cells and were highly similar in profile (Supplementary Figures S3B,C). We speculate that these are mature myelinating oligodendrocytes. Nearly all the OLI3 cells expressed G2M/S-phase cycling genes while OLI4 cells were mostly quiescent and expression genes from the G0/1-phase of the cell cycle (Supplementary Figure S3D). In summary, our data indicate the presence of 2 OPC populations, with one pre-myelinating oligodendrocyte population and two mature oligodendrocyte profiles with similar profiles.

### Single-cell RNA sequencing reveals unique gene signatures for stellate cells and pyramidal neuron populations

**FIGURE 3:**
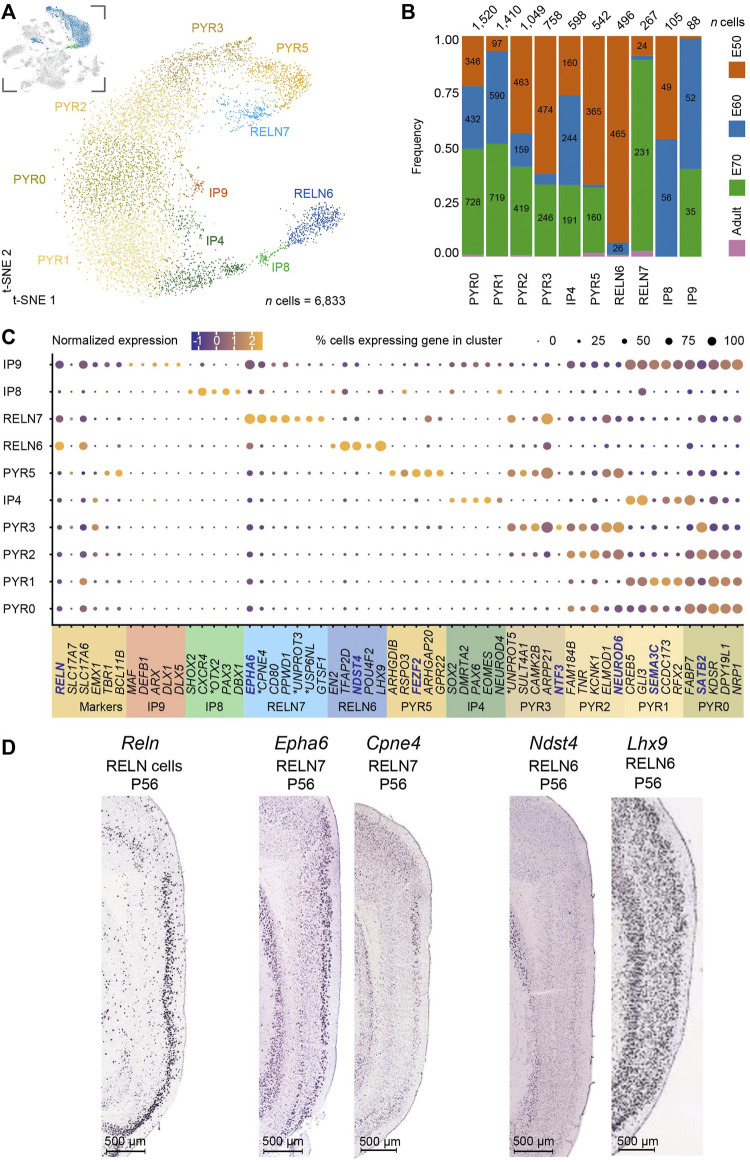
Subclustering of the excitatory neurons and intermediate progenitors (IPs) of the developing medial entorhinal cortex (MEC) highlight two *RELN* + clusters and five pyramidal neuron clusters. **(A)** A t-SNE plot highlights subclustering of excitatory neurons and IPs [number (*n*) of cells = 6,833] results in ten distinct clusters. The inserted image shows the excitatory neuron clusters highlighted in blue and IPs in green, that were subclustered from the parent dataset. **(B)** Distribution of developmental stages within the ten clusters highlights five populations that persist postnatally (three pyramidal neurons, PYR0, PYR2, PYR5 and two Reelin + cell populations, RELN6, RELN7). Three IP populations were identified IP4, IP8 and IP9, with the latter also expressing IN markers and RELN. IP4 was also detected postnatally but expressed IP markers *EOMES*, *PAX6* and *SOX2* **(C)** Unique gene signatures for the ten clusters were determined following differential gene expression analysis. Localization of genes with names in blue are shown in **(D)**. The ∗ marks the human ortholog names (Supplementary Table S4). **(D)** Sagittal sections from the ISH datasets from the Allen Mouse Brain Atlas highlight the location of selected markers from the RELN clusters across the EC layers at P56. Image credit: Allen Institute (2004). Scale bars: 500 μm.

We then focused our attention on the excitatory neurons in order to see if we could identify the stellate cell progenitor and adult cell clusters. First, we performed subclustering on the IP and excitatory neuron populations from the parent dataset, including the excitatory clusters 0, 5, 10, 15, and 17 and the IP cluster 19 (a total of 6,833 cells) Figure 3A, insert). The subclustering revealed ten distinct populations, including three populations exhibiting IP identities (Figure 2A). Cells in cluster IP4 expressed the typical IP markers *EOMES, SOX2*, and *NEUROD4* (Figure 2C) (Pollen et al., 2015) and could be detected across all stages of development, including the postnatal brain, suggestive of ongoing postnatal neurogenesis (Figure 3B). This cluster expressed markers found in the pyramidal (PYR) neuron clusters PYR1, PYR0 and given its transcriptomic profile is likely a progenitor for these pyramidal neuron populations. The second IP cluster, IP8, was detected only during the neurogenesis period (E50-E60) (Liu et al., 2021) and expressed neural progenitor markers including, *PAX3* (Blake and Ziman, 2014), *DBX1* (Lacin et al., 2009) and the ventral midbrain progenitor marker *OTX2* (Puelles et al., 2004). IP8 also clustered closely to RELN6 on the t-SNE plot and could be the progenitor of RELN6 neurons (Figure 3C). A third cluster, IP9 was also only present during pre-natal gestation (mostly at E60-E70) (Figure 3B) and clustered closely to PYR0 and PYR2. It expressed *DLX1*, *DLX5* and *ARX* (Figures 3A–C), together with *GAD1/2* and *RELN* (Figure 3C), suggesting it has a GABAergic IN progenitor-like identity. Despite this, it expressed many genes found in both the RELN7 and PYR clusters (Figure 3C) and might be a progenitor for both the RELN7 cluster and pyramidal neurons*.*

An analysis of canonical genes for neurons with a pyramidal-like identity revealed five clusters. Clusters PYR0, PYR1, PYR2, PYR3 and PYR5 were tangentially located on the t-SNE plot (Figure 3A) and expressed low levels of *RELN* (Figure 3C). Surprisingly, we did not observe in our dataset any neuron populations expressing *CALB1*, a common pyramidal neuron marker reported in rats, mice and humans within the EC (Beall and Lewis, 1992; Diekmann et al., 1994; Fujimaru and Kosaka, 1996). *CALB1* may not be a suitable marker for pyramidal neurons in the pig EC as a previous study has reported the absence of expression in pyramidal neurons in the hippocampal region (Holm et al., 1990). Clusters PYR0 and PYR1 shared similar transcriptional profiles but diverged in *NEUROD1* expression. Clusters PYR2 and PYR3 were also similar but had divergent *ARPP21* expression. Only PYR1 and PYR3 could be detected in the prenatal brain and not in the postnatal brain. Given the similar profiles, it is likely that PYR0, PYR2 and PYR5 are adult populations. This does not explain, however, why all five populations are identified during early development, so we cannot eliminate the possibility that these are different pyramidal neurons. Therefore, we deduce to have captured at least three pyramidal neuron populations found in the postnatal brain. An analysis of mature pyramidal neuron markers only partially confirmed the notion of maturation of PYR1, PYR3 and PYR5, as *SLC17A6/7* (vGLUT1/2 genes) expression was observed in PYR0 and PYR2 but not PYR5 (Figure 3C). Taken together, we have identified five pyramidal neuron subtypes during development and at least three mature pyramidal neuron types in the postnatal MEC.

**FIGURE 4:**
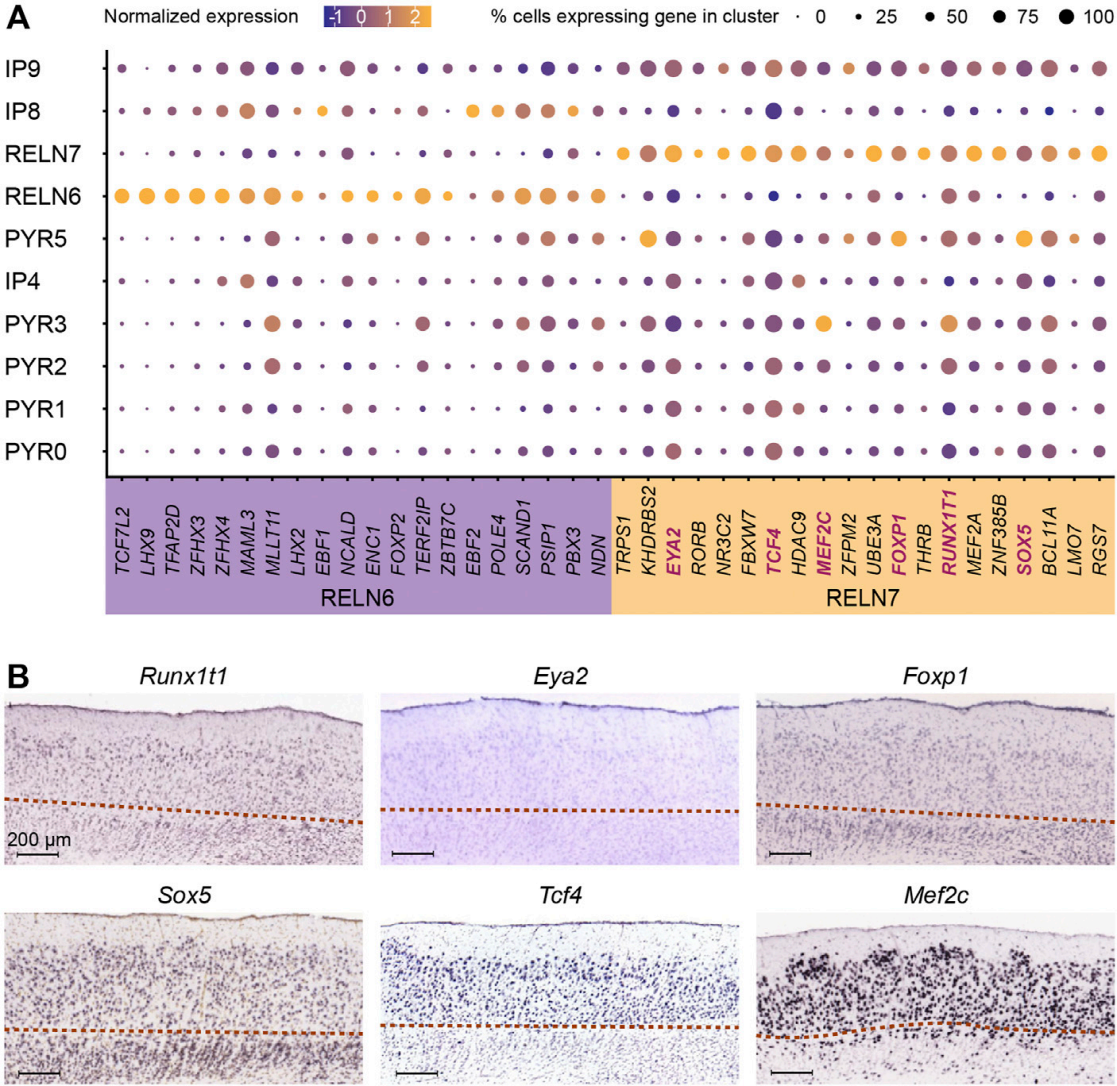
Unique transcriptional factors upregulated in *RELN* positive clusters. **(A)** Expression of the top twenty enriched transcription factors (TFs, x-axis) were identified for each of the *RELN*-positive and putative stellate cell (SC) populations (RELN6, 7) and are visualized across the identified progenitor and excitatory neuron populations (y-axis) in the developing medial entorhinal cortex (MEC). The selected genes for reprogramming induced pluripotent stem cells (iPSCs) are highlighted in blue within the RELN7 cluster. **(B)** The laminar expression of the six stellate cell TFs (*Runx1t1*, *Eya2*, *Foxp1*, *Sox5*, *Tcf4*, and *Mef2c*) was investigated by ISH in sagittal sections of the publicly available Allen Mouse Brain Atlas (Lein et al., 2004; 2007). Image credit: Allen Institute. The red dotted lines demarcate the *lamina dissecans*.

In addition to the pyramidal neurons, the MEC harbors two types of principal neurons expressing Reelin. These are the stellate and intermediate stellate cells that reside in the MEC (Fuchs et al., 2016; Witter et al., 2017). This marker is particularly useful to determine the stellate cell populations from within the excitatory neuron cell types. Our subclustering analysis of the MEC revealed two distinct neuron populations only found in the postnatal brain that expressed *RELN* (RELN 6 and RELN7) (Figures 3A,B). It was important for us to distinguish between these two populations. Apart from the known expression of Reelin and absence of CALB1 (at least in a proportion of Reelin neurons), few other genes are known to be expressed in stellate and intermediate stellate cells (Kitamura et al., 2014; Fuchs et al., 2016; Winterer et al., 2017). Both RELN6 and RELN7 lacked the pyramidal transcription factor, *EMX1* (Chan et al., 2001) (Figure 3C). These two clusters were expressed throughout all sampled time points and persisted in the postnatal adult brain (Figure 3B). They had distinct transcriptional profiles and were clearly separated on the t-SNE plot (Figures 3A,B). In addition, the RELN7 population expressed many markers also observed in the PYR clusters and was positioned closely on the t-SNE with several PYR clusters which might suggest this to be the intermediate stellate cell cluster. Interestingly, RELN6 cells are more abundant than RELN7 cells at E50 and more abundant than the PYR cell types at this time point, suggesting that this may be an early born subtype (Figure 3B). In contrast, more RELN7 cells were captured at E70 [late neurogenesis in the pig (Liu et al., 2021)] than RELN6 cells (Figure 3B). Previous research has shown that the stellate cells are born early on in development, and even earlier than their pyramidal counterparts (Donato et al., 2017) which points to towards the RELN6 cell cluster being these early born stellate cells (Figure 3B). Our previous research in the pig has shown that LII stellate cells co-express BCL11b, SATB2 and Reelin (Liu et al., 2021). Interestingly only the RELN7 cluster co-expressed these genes, whilst RELN6 expressed very low levels of *SATB2*. In the hope to ascertain even more clarity between the two clusters, we investigated the location of the RELN6 and RELN7 populations in the *in-situ* hybridized MEC of the mouse brain from the public databases, the Allen Mouse Brain Atlas, 2004 (Lein et al., 2004; 2007) as a way to identify differences in the positioning of these two clusters. *RELN* expression was unsurprisingly mostly restricted to LII/III of the MEC (Figure 3D). When assessing enriched genes from cluster RELN7 both *Cpne4* and *Epha6* was observed in the LII-LIII in the Allen Mouse Brain Atlas, 2004, confirming the RELN7 neurons were located primarily in the superficial layers (Figure 3). Enriched genes from the RELN6 cluster however were not restricted to LII. For example, both *Lhx9* and *Ndst4* were expressed across all the MEC layers at P56 in the mouse MEC (2004) (Figure 3) which may indicate the location of RELN6 cells are distributed across the MEC layers, however it does not rule out that other cell types expressed these markers in deeper layers. To further dig into unique markers of the RELN populations, we identified differentially expressed transcription factors (TFs) in RELN6 and RELN7 using TFcheckpoint, which is a list of DNA-binding RNA polymerase II TFs curated by Chawla et al. (2013) and by using the Findmarkers tool in Seurat (Figure 4A). Of the many TFs identified, one stood out in the RELN7 cluster, namely *RORB* which has been recently identified as a marker for vulnerable EC excitatory neurons that are lost in AD (Leng et al., 2021). Given the expression of this gene in RELN7 we felt more confident that RELN7 whilst potentially being the intermediate stellate cell cluster, might be the important cell population to target for modeling AD. To sum, the RELN7 cluster appears to be primarily located in LII, has an intermediate stellate cell gene identity and is a relevant cell type for modeling AD given its expression of *RORB*. The IP9 progenitor may be the progenitor for the RELN7 cluster, but further studies would be needed to indeed prove this to be the case. Given the ambiguity in the IP identities, we decided to take a forward programming approach for production of the putative intermediate stellate cell cluster RELN7.

### Induced expression of six transcription factors in human induced pluripotent stem cells results in SC-like progenitors

With focus on the RELN7 cluster, we used our scRNA-seq to identify important cell-specific transcription factors necessary for cell reprogramming. Of the 20 TFs we identified using TFcheckpoint (Figure 4A), we focused on six, *RUNX1T1*, *SOX5*, *FOXP1*, *MEF2C*, *TCF4* and *EYA2* based on a literature search showing prominent roles for these TFs in neurodevelopment and differentiation. Runx1t1 is specifically expressed in neurons and plays a role in hippocampal neuron differentiation (Linqing et al., 2015; Zou et al., 2020), which also lies in close proximity with the EC within the ventral telencephalon and both structures arise from the medial pallium (Abellan et al., 2014). Eya2 is expressed in neural progenitors and is repressed by Foxg1 during corticogenesis, playing an important role in neuronal differentiation (Kumamoto et al., 2013). Foxp1 is co-expressed with Satb2 and detected in LIII-LVa neurons (Hisaoka et al., 2010). It is also implicated in neural stem cell differentiation and neuronal morphogenesis (Li et al., 2015; Braccioli et al., 2017). We recently demonstrated that MEC stellate cells express SATB2 which further raised our interest in FOXP1 (Liu et al., 2021). *SATB2* was also detected in RELN7 (Figure 3C). SOX5 is an important regulator of early-born deep layer neurons (Kwan et al., 2008) and in differentiation of specific corticofugal neuron subtypes (Lai et al., 2008) but may be involved in SC fate given they also express the deep layer marker, BCL11B (Liu et al., 2021). Tcf4 is expressed in cortical and hippocampal neurons, highly enriched in the pallial region (Kim et al., 2020) and is involved in neurogenesis, neuronal differentiation and hippocampal formation (Hill et al., 2017; Mesman et al., 2020; Schoof et al., 2020). It is also implicated in memory and spatial learning (Kennedy et al., 2016). Mef2c plays a role in neuronal development and is expressed in hippocampal neurons (Adachi et al., 2016). It is also implicated in cognitive learning, including working memory and object recognition (Mitchell et al., 2018). An assessment of these genes in the mouse brain ISH data from the Allen Mouse Brain Atlas demonstrated that both *Tcf4* and *Mef2c* are expressed in the superficial layers of the MEC, whereas the remaining genes were expressed across all the layers (2004) (Figure 4B). The sequences of these six TFs were also available from Addgene and cloned into TET-on doxycycline inducible plasmids from either mouse (Foxp1, Sox5) or human (TCF4, MEF2C, RUNX1T1, EYA2) sequences.

**FIGURE 5:**
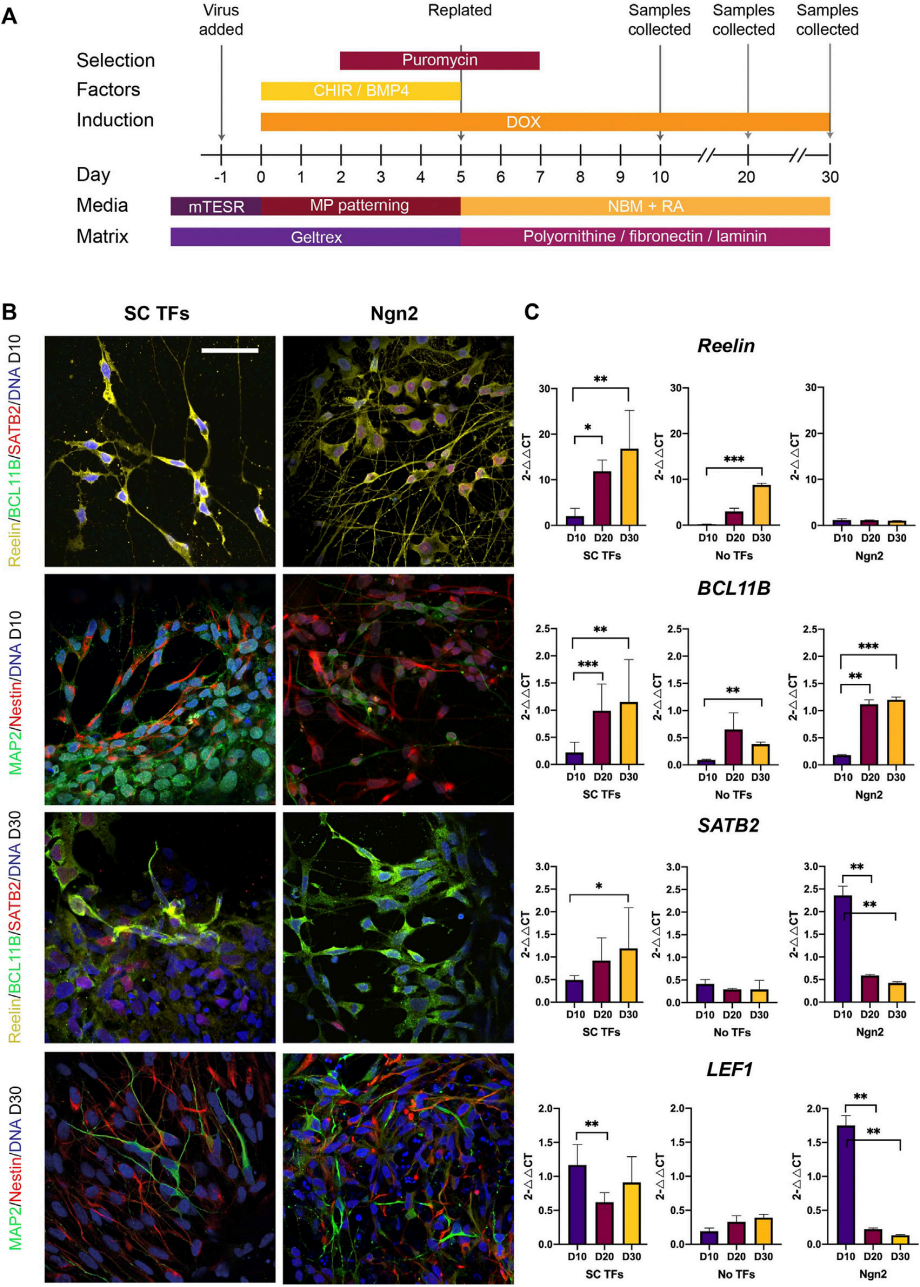
Direct reprogramming of induced pluripotent stem cells (iPSCs) results in the production of stellate cell (SC)-like cells. **(A)** Schematic diagram of the protocol used for directly reprogramming iPSCs into stellate cells using the selected transcription factors (TFs), including timing of media and matrix change, transfection, gene induction (using DOX), addition of patterning factors (CHIR 99021 and BMP4), and collection of samples. Abbreviations: Doxycycline (DOX); pluripotent stem cell media (mTESR); neurobasal medium with medial pallium patterning factors (MP patterning); neurobasal medium for maintenance of neurons (NBM). **(B)** Using immunocytochemistry, iPSCs transduced with the stellate cell transcription factors (SC TFs) result in neurons expressing Reelin at Day (D) 10 which switch on BCL11B and SATB2 at D30 post transduction. iNeurons produced using Ngn2 express Reelin and SATB2 at D10 and by D30 many REELIN + neurons express BCL11B + but only a few express SATB2. Scale bar: 25 μm. **(C)** qPCR at D10, D20 and D30 post transduction shows increasing expression of *RELN*, *BCL11B and SATB2* in iPSCs transduced with SC TFs over time and upregulated expression of *LEF1* as early as D10 in the iPSCs forward programmed with SC TFs. Expression values are fold changes using the equation, 2^△△CT^. CT values were normalized to the housekeeping gene, *ACTIN* and △Ct was normalized to one of the three iPSC lines differentiated with the TFs at either D10 or D30 to form △△Ct. Error bars represent the standard deviation. Statistical t-test was performed between treatment groups and significance is reported as: ∗*p* < 0.05, ∗∗*p* < 0.01, ∗∗∗*p* < 0.001. Experimental N = 3.

In order to produce intermediate stellate cells from human iPSCs, we applied a forward programming approach in combination with culture conditions to pattern the progenitors at an early stage into a medial pallial fate. Evidence suggests the MEC is derived from the medial pallium (Bruce and Neary, 1995; Abellan et al., 2014; Desfilis et al., 2018) and a combination of BMP4 and CHIR 99021 (GSK3 inhibitor and Wnt agonist) can induce human embryonic stem cells into dorsomedial telencephalic tissue (Sakaguchi et al., 2015). As a positive control, we applied a standard forward programming protocol which creates iNeurons (using overexpression of Ngn2). iNeurons have a cortical sensory neuron phenotype (Schornig et al., 2021) and are a standard neuron subtype produced in the stem cell field using forward programming. We performed lentiviral transduction in three human iPSC lines, SFC180-01-01/StBCi064-A, SBAD-03-01 and SBAD-02-01 using six lentiviruses; each containing one of the stellate cell TFs: *Foxp1*, *Sox5 TCF4*, *MEF2C*, *RUNX1T1* or *EYA2* together with the transactivator rtTA lentivirus. As a positive control, iNeurons were produced (also using a TET-on doxycycline inducible system) and cultured in the same culture media as the stellate cell TF treatment. As a negative control, no lentivirus was added. The iPSCs were cultured in 50:50 Neurobasal medium: DMEM/F12 containing GlutaMAX, Glucose, B27 minus Vit A supplement, N2 supplement, ITS-A, NEAA, doxycycline, CHIR and BMP4 for 5 days (Figure 5A). Nb. Doxycycline was not added to the No TF negative control treatment. Puromycin selection was initiated on day (D) 2 in the cells transduced with virus and continued 5 days to eliminate non-transduced cells. The cells were re-seeded on D5 onto triple-coated (poly-L-ornithine/fibronectin/Poly-D laminin) plates and propagated in Neurobasal A medium containing GlutaMAX, B-27, DAPT, pen strep and doxycycline for up to a further 25 days (total 30 days) (Figure 5A).

We first examined expression of the transgenes in the stellate cell TF transduced iPSCs. Increased expression of all six stellate cell TFs was found 10 days following transduction compared to the No TF control (Supplementary Figure S4) indicating that the plasmids could induce expression of the transgenes. Transduction of the 6 SC TFs resulted in the production of cells with neuronal morphology expressing Reelin at D10 (Figure 5B). The cells had a bipolar morphology with long neuronal projections (Figure 5B). Reelin + neurons began co-expressing BCL11B+ and SATB2+ later at D30 suggesting changes in gene expression and possible maturation over time (Figure 5B, Supplementary Figure S5). This was confirmed in qPCR data showing increasing expression of *Reelin*, *BCL11B* and *SATB2* over time (Figure 5C). The iPSC-derived stellate cell like-cells also expressed the hippocampal and MEC marker *LEF1* (Abellan et al., 2014) at D10 and retained expression until D30 (Figure 5C). A mixture of Nestin+ and MAP2+ cells could be detected in the culture both at D10 and D30 (Figure 5B) suggesting a mixture of immature and mature neuronal phenotypes following transduction. Quantification of the proportion of Reelin + neurons at D10 was 25.5% (cells counted = 2,889). At D10, the proportion of Nestin + progenitors and MAP2+ neurons were 60.7 and 54.6%, respectively (cells counted = 5706) with some co-expressing Nestin and MAP2. To sum, the overexpression of the intermediate stellate cell TFs resulted in a mixed culture of progenitors and more mature neurons that expressed the MEC markers Reelin and *LEF1* by D10 and by D30 had switched on/co-expressed BCL11B and SATB2 suggestive of a stellate cell-like cell phenotype. We refer to these cells hereafter, as iPSC-derived stellate cell-like cells.

Interestingly, the iNeurons also expressed Reelin, BCL11B and SATB2 at D10 and retained Reelin and BCL11B expression even at D30 (Figure 5B). *SATB2*/SATB2 expression however, decreased by D20 to low levels (Figures 5B,C), which is similar as previously reported in iNeurons (Miskinyte et al., 2018). However, a few iNeurons co-expressed Reelin, BCL11B and SATB2 at D30 (Figure 5B). The iNeurons upregulated *LEF1* at D10 which, unlike the stellate cell TF treatment, was decreased at D20 and remained low at D30 (Figure 5C). This indicates that the medial pallial inducing cultured conditions had a temporal effect on the expression of iNeurons. In the No TFs control treatment, Reelin, BCL11B or SATB2 could not be detected (Supplementary Figure S6). However, increased levels of *Reelin* and *BCL11B* mRNA were detectable at the later time point of culture (D30) (Figure 5C). Analysis of Nestin and MAP2 expression in the No TF control showed the presence of Nestin + progenitors at D30 and a few MAP2+ neurons (Supplementary Figure S6), further indicating a neutralizing effect of the media, but not specification into entorhinal neuronal subtypes.

**FIGURE 6:**
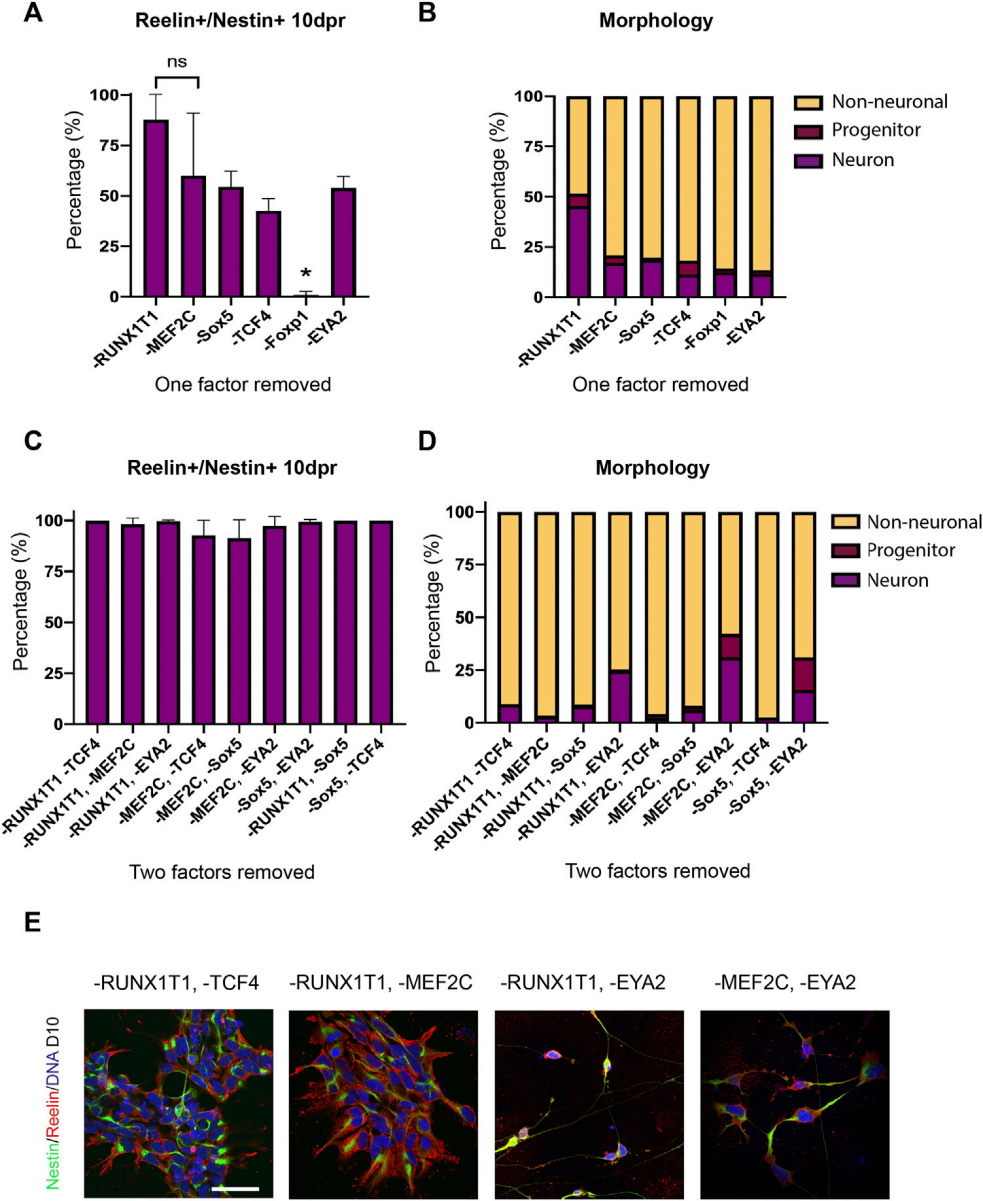
Efficiency and morphology of transduced induced pluripotent stem cells (iPSCs) after dropping out a single transcription factor. **(A)** Co-expression of Reelin and Nestin in cells 10 days post induction (dpi) was significantly decreased when Foxp1 was dropped out (*p* = 0.0013 using a one-way ANOVA) in three human iPSC lines **(B)** Morphology of transduced cells revealed that removal of RUNX1T1 resulted in a higher number of neurons in the SFC180 iPSC line. **(C)** Co-expression of Reelin and Nestin in cells 10 dpi with Foxp1 but with drop out of two factors, showed no significant difference between transcription factor combinations and high conversion rates in the SFC180 iPSC line. **(D)** Assessment of morphology 10 dpi with Foxp1 but drop out of two factors showed loss of EYA2 resulted in higher numbers of neurons indicating EYA2 is redundant in the reprogramming process. **(E)** Immunocytochemistry of forward programmed cells 10 dpi shows that dropping out RUNX1T1 in combination with either TCF4 or MEF2C results in cells co-expressing Nestin and Reelin with neural progenitor morphology, whereas dropping out of EYA2 in combination with RUNX1T1 or MEF2C results in more Nestin+/Reelin + neurons. More neurons can be observed in the -RUNX1T1, -EYA2 and in the -MEF2C, -EYA2 dropout images. Scale bar: 25 μm.

**FIGURE 7:**
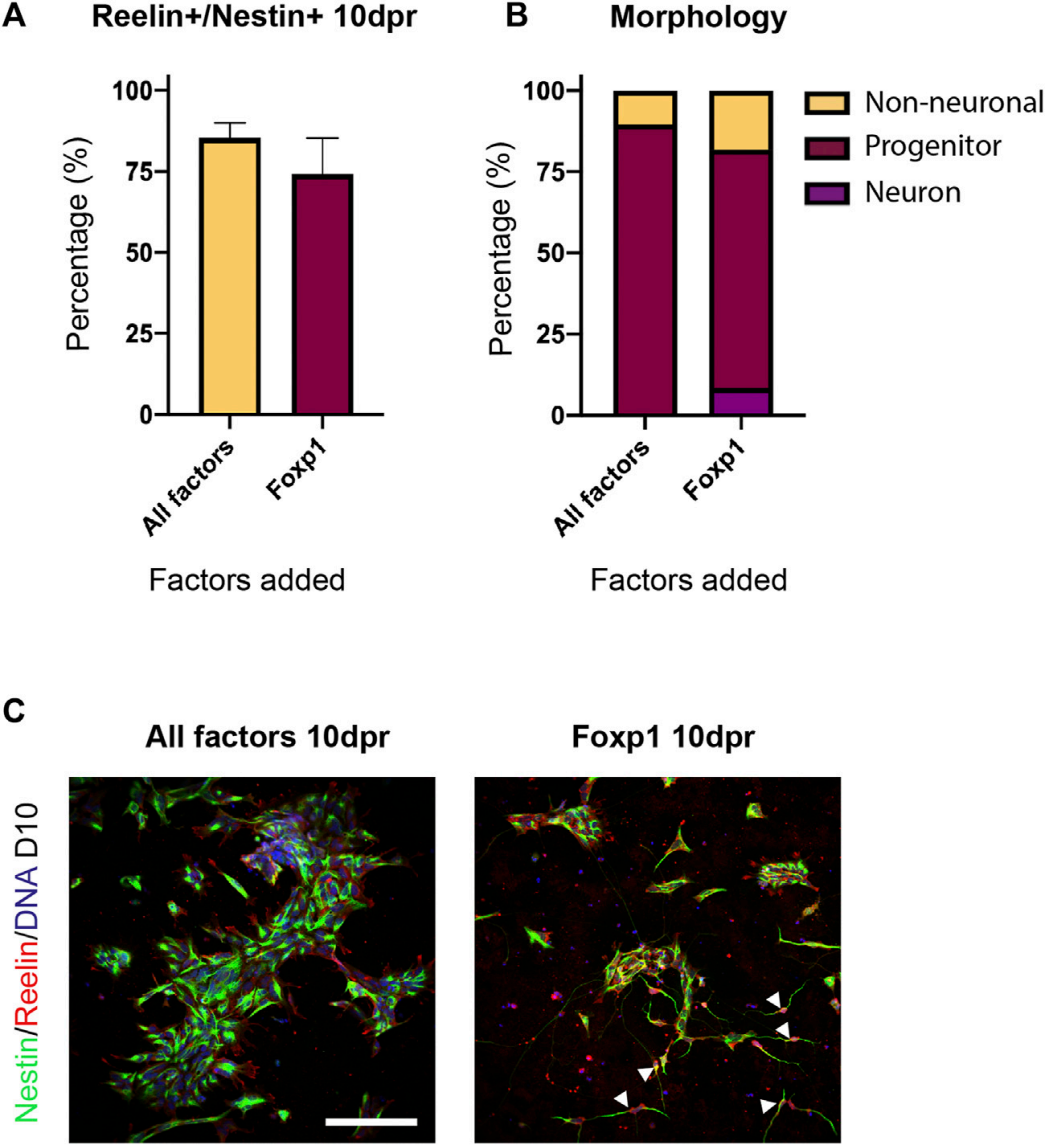
Efficiency and morphology of transduced induced pluripotent stem cells (iPSCS) containing all factors or containing Foxp1 alone. **(A)** Co-expression of Reelin and Nestin in cells 10 days post induction (dpi) was not significantly different between the All-factors treatment or with Foxp1 alone in the SFC180 human iPSC line. Experimental N = 3 **(B)** Morphology of transduced cells revealed a high conversion into Nestin expressing progenitors and neurons in the SFC180 iPSC line and the presence of more neurons when Foxp1 was used alone. **(C)** Immunostaining showing Nestin and Reelin expression in cells 10 dpi in the All-factors combination and Foxp1 alone. White arrowheads highlight neuron morphology. Scale bar: 150 μm.

We then sought to determine if all factors were required in the reprogramming process. We repeated the forward programming protocol using all three cell lines, but this time dropped out a single TF from the total pool of TFs. At D10 following programming we compared the proportion of Reelin+/Nestin + cells between each treatment to determine if differences could be observed in the efficiency in producing Reelin + cells (>500 cells counted/treatment). We found that the removal of Foxp1 from the TF pool resulted in almost no Reelin+/Nestin + cells (mean: 0.95%), whilst the other treatments had significantly higher proportions of Reelin+/Nestin + cells (Figure 6A). This indicated that Foxp1 was an important TF in the reprogramming process. Interestingly, the removal of RUNX1T1 from the TF pool resulted in a significantly higher proportion of Reelin+/Nestin + cells (mean 87.8%) compared to all treatments with the exception of MEF2C (Figure 6A), indicating that RUNX1T1 is likely a redundant factor and potentially even repressing the reprogramming process. Next, we qualitatively evaluated (>300 cells per treatment) the morphology of the cells following the reprogramming process in one human iPSC line (SFC180). We classified cells as either having neuron, progenitor or non-neuronal morphology based on the expression of markers and morphological characteristics. Neurons were classified as having thin elongated neurites with small somas and expressed Nestin. Progenitors were bi-polar elongated cells with thicker cytoplasmic processes expressing Nestin and non-neuronal cells were all other cell shapes that may or may not express Nestin. This qualitative analysis revealed that the TF pool without RUNX1T1 contained more neurons and progenitors than the other TF dropout treatments and confirmed RUNX1T1 is a redundant TF, inhibiting the differentiation process (Figure 6B). We then performed a further drop-two-TF out experiment on the SFC180 cell line in technical triplicates where Foxp1 was retained in all combinations, given its importance, but two further TFs were removed. In this case, no differences in co-expressing Reelin+/Nestin + cells could be observed between the different TF pools 10 days post induction (10 dpi) (Figure 6C). However, morphological analysis of the cell types indicated that removal of the TF EYA2 increased the proportion of neurons and progenitors obtained (Figures 6D,E). The negative effects of RUNX1T1 in the drop two TF out experiments were only evident in the -RUNX1T1, -EYA2 combination, suggesting that the TFs TCF4, MEF2C and Sox5 play contributing roles in the forward programming process. Finally, we tested using the TF Foxp1 alone in forward programming the SFC180 human iPSC line into neurons and compared the outcome to using all SCT TF factors. Here we found both Foxp1 and all the factors resulted in a high expression of co-expressing Reelin+/Nestin + cells 10 dpi (Figure 7A). Morphological assessment of the cells indicated that neurons were observed in the Foxp1 only treatment (8%) already at 10 dpi, but only progenitors were observed in all factor’s combination (Figure 7B). There was also a comparable number of progenitors between all factors (90%) and Foxp1 (73%) only treatment at 10 dpi (Figures 7B,C). These experiments help consolidate the importance of Foxp1 in the forward programming process and that both RUNX1T1 and EYA2 are redundant in the programming process. Together, we have assessed the importance of the TFs in the forward programming process and highlight that Foxp1, Sox2, MEF2C and TCF4 are important TFs in directing differentiation events of human iPSCs into putative intermediate stellate cells.

## Discussion

Our study demonstrates that scRNA-seq data from the developing and postnatal porcine MEC could be used to identify TFs important for differentiation that can be utilized in forward programming of pluripotent stem cells. In this case, we were able to produce putative intermediate stellate cells from human iPSCs that may be functionally relevant for the study of early mechanisms in Alzheimer’s disease. We were able to originally identify six TFs: Foxp1, Sox5*,* TCF4, MEF2C, RUNX1T1 and EYA2 and determined this combination gives rise to putative intermediate stellate cells expressing RELN, BCL11B and SATB2, which is a unique combination of genes that are not observed in other neocortical glutamatergic neurons. In other regions of the neocortex, BCL11B is considered a LV marker (Chen et al., 2008) and SATB2 a superficial layer marker (Slomianka et al., 2011) with RELN being expressed predominantly in both GABAergic interneurons (Pesold et al., 1998) and astrocyte subtypes in the white matter (Hochstim et al., 2008). We have previously identified this unique gene expression combination in neurons located in the murine and porcine MEC superficial layers (Liu et al., 2021) where stellate cells are known to reside (Canto and Witter, 2012). The expression of *LEF1* is also highly enriched in the MEC LII (Ramsden et al., 2015) and in the postnatal medial entorhinal cortex of the Allen Brain Atlas, which suggests these cells have a superficial neuron identity. The production of putative intermediate stellate cells may be of interest for studying the accumulation of amyloid that has been observed in Reelin + neurons within LII of the EC (Kobro-Flatmoen et al., 2016) and mechanisms in early AD. Our characterization using this unique combinational marker approach and expression of these markers in the RELN7 cluster from our scRNAseq data provides an indication for stellate cell specificity. Further studies assessing these neurons using patch-clamping or multi-electrode arrays will help to confirm the functional capacity of these neurons produced *in-vitro*.

Our drop out studies helped to determine that Foxp1 plays a critical role in the reprogramming process. Future studies comparing the transcriptomes of Foxp1 iNeurons vs. SC TF iNeurons will help to assess the similarities between the two neuron populations produced. Foxp1 has previously been shown to be important in the differentiation of neural stem cells (Braccioli et al., 2017) as well as differentiation of neural progenitors into medium spiny neurons (Precious et al., 2016). Previous research also has shown how Foxp1 mediates neurogenesis of deep layer neurons (Pearson et al., 2020). Our research highlights Foxp1 can induce neurons and progenitors from undifferentiated human iPSCs, indicating it may be a master regulator in neuronal differentiation and plays an important role in stellate cell development in the allocortex.

In this study, we assumed the RELN7 cluster to represent intermediate stellate cells and that the RELN6 cluster represents stellate cells based on the clustering analyses and expression of *BCL11b* and *SATB2*. The RELN6 cluster expressed *BCL11B* but was *RORB* negative and expressed only low levels of *SATB2*. Our previous studies in the mouse MEC show Reelin+/Bcl11b + neurons are born in the superficial layers (LII and LIII) from E12.5 to E15, but most are born early on, at E12.5 (Liu et al., 2021). Another subset of Reln+/Bcl11b-neurons was also born from E12.5 to E15 (Liu et al., 2021). Our data here help to justify these two distinct Reelin populations. In our scRNA-seq data, the RELN6 cells arise earlier than the RELN7 cells helping confirm that two separate stellate cell populations are born independently in the developing EC. However, our interest rose in the RELN7 cluster since this cluster expressed *RORB*, which has recently been identified as being expressed in vulnerable neurons in the entorhinal cortex that are lost in AD (Leng et al., 2021). Our data highlights that the Reelin + intermediate stellate cells might be the cell type that is vulnerable in AD over the stellate cells. Future studies using patchseq in AD brain slices could help to define the differences between these two RELN populations and whether the intermediate stellate cell is indeed more vulnerable to the disease than stellate cells.

Interestingly, the positive control iNeurons were found to express Reelin at the protein level at D10 but not at the RNA level. Further, we could identity RNA expression of *BCL11B* at D10 in the SC TFs which was not detected at the protein level. Our explanation for this is that RNA expression is more dynamic and may have degraded quickly. Discrepencies in RNA and protein expression is a common observation in mammalian cells and yeast (Greenbaum et al., 2003; Tian et al., 2004; Wang, 2008). However, consolidation of RNA and protein expression always serves to strengthen confidence in the findings, which in this particular case was not found.

Our study also demonstrates that pig scRNA-seq data can be readily applied for generation of novel protocols in human iPSCs. Indeed, we found the developing MEC dataset projected extremely well using scmap to human and mouse fetal datasets. This was the case, even when the human datasets were at unmatched gestational ages and from the embryonic prefrontal cortex and adult middle temporal gyrus. Our previous findings have also identified that the pig poses as an excellent model of neurogenesis, given its long gestational length, completion of corticogenesis prior to birth and presence of a moderate outer subventricular zone (Liu et al., 2021). Our single-cell profiling of the porcine postnatal MEC revealed a high-quality dataset which corroborates and further enhances knowledge of MEC cell types in the developing and postnatal brain. Our dataset on the MEC resulted in a mean of 2,798 different genes captured per cell in the 24,294 cells. Our dataset includes many glia which we believe was a result from dissecting more white matter than grey matter. Nevertheless, the dataset includes many MEC neurons which could be annotated using subtype-specific markers. Classification of the dataset allowed us to identify at least three subtypes of pyramidal neurons, two stellate cell subtypes and three IP populations in the postnatal MEC. We were also able to detect six IN progenitor populations. In regions where IN diversity have been well studied such as the amygdala, at least six classes of INs have been identified based on their discharge properties and electrophysiological profiles in the basolateral amygdala (Polepalli et al., 2020) and similar research is required in the EC to fully differentiate between the different IN subclasses. The INs lying in the superficial layers are particularly interesting for researchers studying the mechanisms underlying grid cell firing. We also confer with the study, that two oligodendrocyte populations exist in the postnatal brain Leng et al. (2021). Finally, we saw very little overlap in excitatory neuron subtypes (0.3%) when we projected our dataset to the human temporal cortex. Only a small number of excitatory neurons projected to human temporal cortex neurons belonging to the L5-6 Fzef2_Tle4 subfamily, the L4 Rorb_Mme and the L2 Cux2_Prss12 glutamatergic subtypes suggesting excitatory neurons are unique compared to other regional areas. This is in concordance to Franjic et al., which found the EC excitatory neurons were distinct from excitatory neurons residing in close brain regions including the CA regions and subiculum. Our dataset and comparisons to other datasets therefore provides excellent insight into the uniqueness and cell diversity of the developing and adult MEC.

In conclusion, we report for the first time, transcriptomic data of cell types uniquely found in the MEC and use a forward programming approach by overexpressing Foxp1, Sox5, TCF4, MEF2C, RUNX1T1 and EYA2 to generate putative intermediate stellate cell-like cells from human iPSCs. We identify that Foxp1 acts as a master regulator to induce neurons and progenitors from undifferentiated iPSCs and plays an important role in the generation of intermediate stellate cells in the EC. In contrast, EYA2 and RUNX1T1 were not essential in the forward programming process. A combination of all six factors resulted in neurons with a unique intermediate stellate cell identity. Future studies focused on studying relevant neurons affected early on in disease in regions such as the entorhinal cortex will help to more accurately determine early mechanisms of the disease. We envision that the generation and study of iPSC-derived intermediate stellate cells from AD patients will be particularly relevant models for investigation.

## Data Availability

The datasets generated are available at the NCBI repository with GEO accession number GSE134482 (https://www.ncbi.nlm.nih.gov/geo/query/acc.cgi?acc=GSE134482). The submitted data includes the raw sequencing data as fastq files together with the processed count matrix used in this study. All R code used for the analysis of this study is publicly available at github: https://github.com/BDD-lab/EC-scRNAseq-2021.

## References

[B1] Abellan A. Desfilis E. Medina L. (2014). Combinatorial expression of Lef1, Lhx2, Lhx5, Lhx9, Lmo3, Lmo4, and Prox1 helps to identify comparable subdivisions in the developing hippocampal formation of mouse and chicken. Front. Neuroanat. 8, 59. 10.3389/fnana.2014.00059 25071464 PMC4082316

[B2] Adachi M. Lin P. Y. Pranav H. Monteggia L. M. (2016). Postnatal loss of Mef2c results in dissociation of effects on synapse number and learning and memory. Biol. Psychiatry 80, 140–148. 10.1016/j.biopsych.2015.09.018 26642739 PMC4826326

[B3] Allen Mouse Brain Atlas (2004). Allen Institute for brain science. Washington, DC: Allen Mouse Brain Atlas [Online]. Available at: https://mouse.brain-map.org/.[Accessed]

[B4] Arnold S. E. Hyman B. T. VAN Hoesen G. W. Damasio A. R. (1991). Some cytoarchitectural abnormalities of the entorhinal cortex in schizophrenia. Arch. Gen. Psychiatry 48, 625–632. 10.1001/archpsyc.1991.01810310043008 2069493

[B5] Artegiani B. Lyubimova A. Muraro M. VAN Es J. H. VAN Oudenaarden A. Clevers H. (2017). A single-cell RNA sequencing study reveals cellular and molecular dynamics of the hippocampal neurogenic niche. Cell Rep. 21, 3271–3284. 10.1016/j.celrep.2017.11.050 29241552

[B6] Beall M. J. Lewis D. A. (1992). Heterogeneity of layer II neurons in human entorhinal cortex. J. Comp. Neurol. 321, 241–266. 10.1002/cne.903210206 1500542

[B7] Blake J. A. Ziman M. R. (2014). Pax genes: Regulators of lineage specification and progenitor cell maintenance. Development 141, 737–751. 10.1242/dev.091785 24496612

[B8] Blondel V. D. Guillaume J.-L. Lambiotte R. Lefebvre E. (2008). Fast unfolding of communities in large networks. J. Stat. Mech. 1, P10008.

[B9] Braccioli L. Vervoort S. J. Adolfs Y. Heijnen C. J. Basak O. Pasterkamp R. J. (2017). FOXP1 promotes embryonic neural stem cell differentiation by repressing Jagged1 expression. Stem Cell Rep. 9, 1530–1545. 10.1016/j.stemcr.2017.10.012 PMC5688236 29141232

[B10] Bruce L. L. Neary T. J. (1995). The limbic system of tetrapods: A comparative analysis of cortical and amygdalar populations. Brain Behav. Evol. 46, 224–234. 10.1159/000113276 8564465

[B11] Butler A. Hoffman P. Smibert P. Papalexi E. Satija R. (2018). Integrating single-cell transcriptomic data across different conditions, technologies, and species. Nat. Biotechnol. 36, 411–420. 10.1038/nbt.4096 29608179 PMC6700744

[B12] Canto C. B. Witter M. P. (2012). Cellular properties of principal neurons in the rat entorhinal cortex. II. The medial entorhinal cortex. Hippocampus 22, 1277–1299. 10.1002/hipo.20993 22161956

[B13] Chan C. H. Godinho L. N. Thomaidou D. Tan S. S. Gulisano M. Parnavelas J. G. (2001). Emx1 is a marker for pyramidal neurons of the cerebral cortex. Cereb. Cortex 11, 1191–1198. 10.1093/cercor/11.12.1191 11709490

[B14] Chawla K. Tripathi S. Thommesen L. Laegreid A. Kuiper M. (2013). TFcheckpoint: A curated compendium of specific DNA-binding RNA polymerase II transcription factors. Bioinformatics 29, 2519–2520. 10.1093/bioinformatics/btt432 23933972

[B15] Chen B. Wang S. S. Hattox A. M. Rayburn H. Nelson S. B. Mcconnell S. K. (2008). The Fezf2-Ctip2 genetic pathway regulates the fate choice of subcortical projection neurons in the developing cerebral cortex. Proc. Natl. Acad. Sci. U. S. A. 105, 11382–11387. 10.1073/pnas.0804918105 18678899 PMC2495013

[B16] Desfilis E. Abellan A. Sentandreu V. Medina L. (2018). Expression of regulatory genes in the embryonic brain of a lizard and implications for understanding pallial organization and evolution. J. Comp. Neurol. 526, 166–202. 10.1002/cne.24329 28891227 PMC5765483

[B17] Diekmann S. Nitsch R. Ohm T. G. (1994). The organotypic entorhinal-hippocampal complex slice culture of adolescent rats. A model to study transcellular changes in a circuit particularly vulnerable in neurodegenerative disorders. J. Neural Transm. Suppl. 44, 61–71. 10.1007/978-3-7091-9350-1_5 7897400

[B18] Dobin A. Davis C. A. Schlesinger F. Drenkow J. Zaleski C. Jha S. (2013). Star: Ultrafast universal RNA-seq aligner. Bioinformatics 29, 15–21. 10.1093/bioinformatics/bts635 23104886 PMC3530905

[B19] Donato F. Jacobsen R. I. Moser M. B. Moser E. I. (2017). Stellate cells drive maturation of the entorhinal-hippocampal circuit. Science 355, eaai8178. 10.1126/science.aai8178 28154241

[B20] Franjic D. Skarica M. Ma S. Arellano J. I. Tebbenkamp A. T. N. Choi J. (2021). Transcriptomic taxonomy and neurogenic trajectories of adult human, macaque, and pig hippocampal and entorhinal cells. Neuron 110, 452–469.e14. 10.1016/j.neuron.2021.10.036 34798047 PMC8813897

[B21] Fuchs E. C. Neitz A. Pinna R. Melzer S. Caputi A. Monyer H. (2016). Local and distant input controlling excitation in layer II of the medial entorhinal cortex. Neuron 89, 194–208. 10.1016/j.neuron.2015.11.029 26711115 PMC4712190

[B22] Fujimaru Y. Kosaka T. (1996). The distribution of two calcium binding proteins, calbindin D-28K and parvalbumin, in the entorhinal cortex of the adult mouse. Neurosci. Res. 24, 329–343. 10.1016/0168-0102(95)01008-4 8861103

[B23] Gatome C. W. Slomianka L. Lipp H. P. Amrein I. (2010). Number estimates of neuronal phenotypes in layer II of the medial entorhinal cortex of rat and mouse. Neuroscience 170, 156–165. 10.1016/j.neuroscience.2010.06.048 20600643

[B24] Gill K. P. Denham M. (2020). Optimized transgene delivery using third-generation lentiviruses. Curr. Protoc. Mol. Biol. 133, e125. 10.1002/cpmb.125 32986282 PMC7583475

[B25] Gomez-Isla T. Price J. L. Mckeel D. W. JR. Morris J. C. Growdon J. H. Hyman B. T. (1996). Profound loss of layer II entorhinal cortex neurons occurs in very mild Alzheimer's disease. J. Neurosci. 16, 4491–4500. 10.1523/jneurosci.16-14-04491.1996 8699259 PMC6578866

[B26] Greenbaum D. Colangelo C. Williams K. Gerstein M. (2003). Comparing protein abundance and mRNA expression levels on a genomic scale. Genome Biol. 4, 117. 10.1186/gb-2003-4-9-117 12952525 PMC193646

[B27] Grubman A. Chew G. Ouyang J. F. Sun G. Choo X. Y. Mclean C. (2019). A single-cell atlas of entorhinal cortex from individuals with Alzheimer's disease reveals cell-type-specific gene expression regulation. Nat. Neurosci. 22, 2087–2097. 10.1038/s41593-019-0539-4 31768052

[B28] Haghverdi L. Lun A. T. L. Morgan M. D. Marioni J. C. (2018). Batch effects in single-cell RNA-sequencing data are corrected by matching mutual nearest neighbors. Nat. Biotechnol. 36, 421–427. 10.1038/nbt.4091 29608177 PMC6152897

[B29] Henderson V. W. Mack W. Williams B. W. (1989). Spatial disorientation in Alzheimer's disease. Arch. Neurol. 46, 391–394. 10.1001/archneur.1989.00520400045018 2705898

[B30] Hill M. J. Killick R. Navarrete K. Maruszak A. Mclaughlin G. M. Williams B. P. (2017). Knockdown of the schizophrenia susceptibility gene TCF4 alters gene expression and proliferation of progenitor cells from the developing human neocortex. J. Psychiatry Neurosci. 42, 181–188. 10.1503/jpn.160073 27689884 PMC5403663

[B31] Hisaoka T. Nakamura Y. Senba E. Morikawa Y. (2010). The forkhead transcription factors, Foxp1 and Foxp2, identify different subpopulations of projection neurons in the mouse cerebral cortex. Neuroscience 166, 551–563. 10.1016/j.neuroscience.2009.12.055 20040367

[B32] Hochstim C. Deneen B. Lukaszewicz A. Zhou Q. Anderson D. J. (2008). Identification of positionally distinct astrocyte subtypes whose identities are specified by a homeodomain code. Cell 133, 510–522. 10.1016/j.cell.2008.02.046 18455991 PMC2394859

[B33] Hodge R. D. Bakken T. E. Miller J. A. Smith K. A. Barkan E. R. Graybuck L. T. (2019). Conserved cell types with divergent features in human versus mouse cortex. Nature 573, 61–68. 10.1038/s41586-019-1506-7 31435019 PMC6919571

[B34] Holm I. E. Geneser F. A. Zimmer J. Baimbridge K. G. (1990). Immunocytochemical demonstration of the calcium-binding proteins calbindin-D 28k and parvalbumin in the subiculum, hippocampus and dentate area of the domestic pig. Prog. Brain Res. 83, 85–97. 10.1016/s0079-6123(08)61243-1 2392572

[B35] Hu J. G. Wang Y. X. Zhou J. S. Chen C. J. Wang F. C. Li X. W. (2011). Differential gene expression in oligodendrocyte progenitor cells, oligodendrocytes and type II astrocytes. Tohoku J. Exp. Med. 223, 161–176. 10.1620/tjem.223.161 21372517

[B36] Humphray S. J. Scott C. E. Clark R. Marron B. Bender C. Camm N. (2007). A high utility integrated map of the pig genome. Genome Biol. 8, R139. 10.1186/gb-2007-8-7-r139 17625002 PMC2323232

[B37] Kanatani S. Yozu M. Tabata H. Nakajima K. (2008). COUP-TFII is preferentially expressed in the caudal ganglionic eminence and is involved in the caudal migratory stream. J. Neurosci. 28, 13582–13591. 10.1523/JNEUROSCI.2132-08.2008 19074032 PMC6671763

[B38] Kennedy A. J. Rahn E. J. Paulukaitis B. S. Savell K. E. Kordasiewicz H. B. Wang J. (2016). Tcf4 regulates synaptic plasticity, DNA methylation, and memory function. Cell Rep. 16, 2666–2685. 10.1016/j.celrep.2016.08.004 27568567 PMC5710002

[B39] Kim H. Berens N. C. Ochandarena N. E. Philpot B. D. (2020). Region and cell type distribution of TCF4 in the postnatal mouse brain. Front. Neuroanat. 14, 42. 10.3389/fnana.2020.00042 32765228 PMC7379912

[B40] Kiselev V. Y. Yiu A. Hemberg M. (2018). scmap: projection of single-cell RNA-seq data across data sets. Nat. Methods 15, 359–362. 10.1038/nmeth.4644 29608555

[B41] Kitamura T. Pignatelli M. Suh J. Kohara K. Yoshiki A. Abe K. (2014). Island cells control temporal association memory. Science 343, 896–901. 10.1126/science.1244634 24457215 PMC5572219

[B42] Kobro-Flatmoen A. Nagelhus A. Witter M. P. (2016). Reelin-immunoreactive neurons in entorhinal cortex layer II selectively express intracellular amyloid in early Alzheimer's disease. Neurobiol. Dis. 93, 172–183. 10.1016/j.nbd.2016.05.012 27195475

[B43] Kordower J. H. Chu Y. Stebbins G. T. Dekosky S. T. Cochran E. J. Bennett D. (2001). Loss and atrophy of layer II entorhinal cortex neurons in elderly people with mild cognitive impairment. Ann. Neurol. 49, 202–213. 10.1002/1531-8249(20010201)49:2<202::aid-ana40>3.0.co;2-3 11220740

[B44] Krishnaswami S. R. Grindberg R. V. Novotny M. Venepally P. Lacar B. Bhutani K. (2016). Using single nuclei for RNA-seq to capture the transcriptome of postmortem neurons. Nat. Protoc. 11, 499–524. 10.1038/nprot.2016.015 26890679 PMC4941947

[B45] Kumamoto T. Toma K. Gunadimckenna W. L. Kasukawa T. Katzman S. Chen B. (2013). Foxg1 coordinates the switch from nonradially to radially migrating glutamatergic subtypes in the neocortex through spatiotemporal repression. Cell Rep. 3, 931–945. 10.1016/j.celrep.2013.02.023 23523356 PMC3648982

[B46] Kwan K. Y. Lam M. M. Krsnik Z. Kawasawa Y. I. Lefebvre V. Sestan N. (2008). SOX5 postmitotically regulates migration, postmigratory differentiation, and projections of subplate and deep-layer neocortical neurons. Proc. Natl. Acad. Sci. U. S. A. 105, 16021–16026. 10.1073/pnas.0806791105 18840685 PMC2572944

[B47] Lacin H. Zhu Y. Wilson B. A. Skeath J. B. (2009). Dbx mediates neuronal specification and differentiation through cross-repressive, lineage-specific interactions with eve and hb9. Development 136, 3257–3266. 10.1242/dev.037242 19710170 PMC2739143

[B48] Lai T. Jabaudon D. Molyneaux B. J. Azim E. Arlotta P. Menezes J. R. (2008). SOX5 controls the sequential generation of distinct corticofugal neuron subtypes. Neuron 57, 232–247. 10.1016/j.neuron.2007.12.023 18215621

[B49] Lee J. T. H. Patikas N. Kiselev V. Y. Hemberg M. (2021). Fast searches of large collections of single-cell data using scfind. Nat. Methods 18, 262–271. 10.1038/s41592-021-01076-9 33649586 PMC7116898

[B50] Lein E. S. Hawrylycz M. J. Ao N. Ayres M. Bensinger A. Bernard A. (2007). Genome-wide atlas of gene expression in the adult mouse brain. Nature 445, 168–176. 10.1038/nature05453 17151600

[B93] Lein E. S. Zhao X. Gage F. H. (2004). Defining a molecular atlas of the hippocampus using DNA microarrays and high-throughput in situ hybridization. J Neurosci. 24, 3874–3879. 10.1523/JNEUROSCI.4710-03.2004 PMC6729356 15084669

[B51] Leng K. Li E. Eser R. Piergies A. Sit R. Tan M. (2021). Molecular characterization of selectively vulnerable neurons in Alzheimer's disease. Nat. Neurosci. 24, 276–287. 10.1038/s41593-020-00764-7 33432193 PMC7854528

[B52] Li X. Xiao J. Frohlich H. Tu X. Li L. Xu Y. (2015). Foxp1 regulates cortical radial migration and neuronal morphogenesis in developing cerebral cortex. PLoS One 10, e0127671. 10.1371/journal.pone.0127671 26010426 PMC4444005

[B53] Lin G. Mela A. Guilfoyle E. M. Goldman J. E. (2009). Neonatal and adult O4(+) oligodendrocyte lineage cells display different growth factor responses and different gene expression patterns. J. Neurosci. Res. 87, 3390–3402. 10.1002/jnr.22065 19360905 PMC2760623

[B54] Linqing Z. Guohua J. Haoming L. Xuelei T. Jianbing Q. Meiling T. (2015). Runx1t1 regulates the neuronal differentiation of radial glial cells from the rat hippocampus. Stem Cells Transl. Med. 4, 110–116. 10.5966/sctm.2014-0158 25473084 PMC4275013

[B55] Liu Y. Bergmann T. Mori Y. Peralvo Vidal J. M. Pihl M. Vasistha N. A. (2021). Development of the entorhinal cortex occurs via parallel lamination during neurogenesis. Front. Neuroanat. 15, 663667. 10.3389/fnana.2021.663667 34025365 PMC8139189

[B56] Lujan E. Wernig M. (2013). An indirect approach to generating specific human cell types. Nat. Methods 10, 44–45. 10.1038/nmeth.2325 23269377 PMC4109807

[B57] Magri L. Gacias M. Wu M. Swiss V. A. Janssen W. G. Casaccia P. (2014). c-Myc-dependent transcriptional regulation of cell cycle and nucleosomal histones during oligodendrocyte differentiation. Neuroscience 276, 72–86. 10.1016/j.neuroscience.2014.01.051 24502923 PMC4294794

[B58] Mesman S. Bakker R. Smidt M. P. (2020). Tcf4 is required for correct brain development during embryogenesis. Mol. Cell. Neurosci. 106, 103502. 10.1016/j.mcn.2020.103502 32474139

[B59] Miskinyte G. Gronning Hansen M. Monni E. Lam M. Bengzon J. Lindvall O. (2018). Transcription factor programming of human ES cells generates functional neurons expressing both upper and deep layer cortical markers. PLoS One 13, e0204688. 10.1371/journal.pone.0204688 30307948 PMC6181302

[B60] Mitchell A. C. Javidfar B. Pothula V. Ibi D. Shen E. Y. Peter C. J. (2018). MEF2C transcription factor is associated with the genetic and epigenetic risk architecture of schizophrenia and improves cognition in mice. Mol. Psychiatry 23, 123–132. 10.1038/mp.2016.254 28115742 PMC5966823

[B61] Ohmi K. Zhao H. Z. Neufeld E. F. (2011). Defects in the medial entorhinal cortex and dentate gyrus in the mouse model of Sanfilippo syndrome type B. PLoS One 6, e27461. 10.1371/journal.pone.0027461 22096577 PMC3212581

[B62] Olton D. S. Walker J. A. Wolf W. A. (1982). A disconnection analysis of hippocampal function. Brain Res. 233, 241–253. 10.1016/0006-8993(82)91200-8 7059809

[B63] Pastoll H. Garden D. L. Papastathopoulos I. Surmeli G. Nolan M. F. (2020). Inter- and intra-animal variation in the integrative properties of stellate cells in the medial entorhinal cortex. Elife 9, e52258. 10.7554/eLife.52258 32039761 PMC7067584

[B64] Pearson C. A. Moore D. M. Tucker H. O. Dekker J. D. Hu H. Miquelajauregui A. (2020). Foxp1 regulates neural stem cell self-renewal and bias toward deep layer cortical fates. Cell Rep. 30, 1964–1981. e3. 10.1016/j.celrep.2020.01.034 32049024 PMC8397815

[B65] Perez-Garcia C. G. Gonzalez-Delgado F. J. Suarez-Sola M. L. Castro-Fuentes R. Martin-Trujillo J. M. Ferres-Torres R. (2001). Reelin-immunoreactive neurons in the adult vertebrate pallium. J. Chem. Neuroanat. 21, 41–51. 10.1016/s0891-0618(00)00104-6 11173219

[B66] Pesold C. Impagnatiello F. Pisu M. G. Uzunov D. P. Costa E. Guidotti A. (1998). Reelin is preferentially expressed in neurons synthesizing gamma-aminobutyric acid in cortex and hippocampus of adult rats. Proc. Natl. Acad. Sci. U. S. A. 95, 3221–3226. 10.1073/pnas.95.6.3221 9501244 PMC19723

[B67] Pfisterer U. Petukhov V. Demharter S. Meichsner J. Thompson J. J. Batiuk M. Y. (2020). Identification of epilepsy-associated neuronal subtypes and gene expression underlying epileptogenesis. Nat. Commun. 11, 5038. 10.1038/s41467-020-18752-7 33028830 PMC7541486

[B68] Polepalli J. S. Gooch H. Sah P. (2020). Diversity of interneurons in the lateral and basal amygdala. NPJ Sci. Learn. 5, 10. 10.1038/s41539-020-0071-z 32802405 PMC7400739

[B69] Pollen A. A. Nowakowski T. J. Chen J. Retallack H. Sandoval-Espinosa C. Nicholas C. R. (2015). Molecular identity of human outer radial glia during cortical development. Cell 163, 55–67. 10.1016/j.cell.2015.09.004 26406371 PMC4583716

[B70] Precious S. V. Kelly C. M. Reddington A. E. Vinh N. N. Stickland R. C. Pekarik V. (2016). FoxP1 marks medium spiny neurons from precursors to maturity and is required for their differentiation. Exp. Neurol. 282, 9–18. 10.1016/j.expneurol.2016.05.002 27154297 PMC4920670

[B71] Puelles E. Annino A. Tuorto F. Usiello A. Acampora D. Czerny T. (2004). Otx2 regulates the extent, identity and fate of neuronal progenitor domains in the ventral midbrain. Development 131, 2037–2048. 10.1242/dev.01107 15105370

[B72] Ramsden H. L. Surmeli G. Mcdonagh S. G. Nolan M. F. (2015). Laminar and dorsoventral molecular organization of the medial entorhinal cortex revealed by large-scale anatomical analysis of gene expression. PLoS Comput. Biol. 11, e1004032. 10.1371/journal.pcbi.1004032 25615592 PMC4304787

[B73] Rowland D. C. Obenhaus H. A. Skytoen E. R. Zhang Q. Kentros C. G. Moser E. I. (2018). Functional properties of stellate cells in medial entorhinal cortex layer II. Elife 7, e36664. 10.7554/eLife.36664 30215597 PMC6140717

[B74] Sakaguchi H. Kadoshima T. Soen M. Narii N. Ishida Y. Ohgushi M. (2015). Generation of functional hippocampal neurons from self-organizing human embryonic stem cell-derived dorsomedial telencephalic tissue. Nat. Commun. 6, 8896. 10.1038/ncomms9896 26573335 PMC4660208

[B75] Satija R. Farrell J. A. Gennert D. Schier A. F. Regev A. (2015). Spatial reconstruction of single-cell gene expression data. Nat. Biotechnol. 33, 495–502. 10.1038/nbt.3192 25867923 PMC4430369

[B76] Schoof M. Hellwig M. Harrison L. Holdhof D. Lauffer M. C. Niesen J. (2020). The basic helix-loop-helix transcription factor TCF4 impacts brain architecture as well as neuronal morphology and differentiation. Eur. J. Neurosci. 51, 2219–2235. 10.1111/ejn.14674 31919899

[B77] Schornig M. Ju X. Fast L. Ebert S. Weigert A. Kanton S. (2021). Comparison of induced neurons reveals slower structural and functional maturation in humans than in apes. Elife 10, e59323. 10.7554/eLife.59323 33470930 PMC7870144

[B78] Slomianka L. Amrein I. Knuesel I. Sorensen J. C. Wolfer D. P. (2011). Hippocampal pyramidal cells: The reemergence of cortical lamination. Brain Struct. Funct. 216, 301–317. 10.1007/s00429-011-0322-0 21597968 PMC3197924

[B79] Sousa-Pinto A. (1973). The structure of the first auditory cortex (A I) in the cat. I.--Light microscopic observations on its organization. Arch. Ital. Biol. 111, 112–137. 18843819

[B80] Tasic B. Yao Z. Graybuck L. T. Smith K. A. Nguyen T. N. Bertagnolli D. (2018). Shared and distinct transcriptomic cell types across neocortical areas. Nature 563, 72–78. 10.1038/s41586-018-0654-5 30382198 PMC6456269

[B81] Thomsen R. Solvsten C. A. Linnet T. E. Blechingberg J. Nielsen A. L. (2010). Analysis of qPCR data by converting exponentially related Ct values into linearly related X0 values. J. Bioinform. Comput. Biol. 8, 885–900. 10.1142/s0219720010004963 20981893

[B82] Tian Q. Stepaniants S. B. Mao M. Weng L. Feetham M. C. Doyle M. J. (2004). Integrated genomic and proteomic analyses of gene expression in Mammalian cells. Mol. Cell. Proteomics 3, 960–969. 10.1074/mcp.M400055-MCP200 15238602

[B83] Tirosh I. Izar B. Prakadan S. M. Wadsworth M. H., 2N. D. Treacy D. Trombetta J. J. (2016). Dissecting the multicellular ecosystem of metastatic melanoma by single-cell RNA-seq. Science 352, 189–196. 10.1126/science.aad0501 27124452 PMC4944528

[B84] Van Der Maaten L. Hinton G. (2008). Visualizing data using t-SNE. J. Mach. Learn. Res. 9, 2579–2605.

[B85] Wang D. (2008). Discrepancy between mRNA and protein abundance: Insight from information retrieval process in computers. Comput. Biol. Chem. 32, 462–468. 10.1016/j.compbiolchem.2008.07.014 18757239 PMC2637108

[B86] Warr A. Affara N. Aken B. Beiki H. Bickhart D. M. Billis K. (2020). An improved pig reference genome sequence to enable pig genetics and genomics research. Gigascience 9, giaa051. 10.1093/gigascience/giaa051 32543654 PMC7448572

[B87] Wegiel J. Kuchna I. Nowicki K. Imaki H. Wegiel J. Marchi E. (2010). The neuropathology of autism: Defects of neurogenesis and neuronal migration, and dysplastic changes. Acta Neuropathol. 119, 755–770. 10.1007/s00401-010-0655-4 20198484 PMC2869041

[B88] Wigstrom H. (1977). Spatial propagation of associations in a cortex-like neural network model. J. Neurosci. Res. 3, 301–319. 10.1002/jnr.490030409 615279

[B89] Winterer J. Maier N. Wozny C. Beed P. Breustedt J. Evangelista R. (2017). Excitatory microcircuits within superficial layers of the medial entorhinal cortex. Cell Rep. 19, 1110–1116. 10.1016/j.celrep.2017.04.041 28494861

[B90] Witter M. P. Doan T. P. Jacobsen B. Nilssen E. S. Ohara S. (2017). Architecture of the entorhinal cortex A review of entorhinal anatomy in rodents with some comparative notes. Front. Syst. Neurosci. 11, 46. 10.3389/fnsys.2017.00046 28701931 PMC5488372

[B91] Zhong S. Zhang S. Fan X. Wu Q. Yan L. Dong J. (2018). A single-cell RNA-seq survey of the developmental landscape of the human prefrontal cortex. Nature 555, 524–528. 10.1038/nature25980 29539641

[B92] Zou L. Li H. Han X. Qin J. Song G. (2020). Runx1t1 promotes the neuronal differentiation in rat hippocampus. Stem Cell Res. Ther. 11, 160. 10.1186/s13287-020-01667-x 32321587 PMC7178948

