# Supplementary figures and images for: Production of human entorhinal stellate cell-like cells by forward programming shows an important role of Foxp1 in reprogramming
Source: Front Cell Dev Biol. 2022 Aug 15;10:976549. doi: 10.3389/fcell.2022.976549 (PMC9420913; doi:10.3389/fcell.2022.976549)

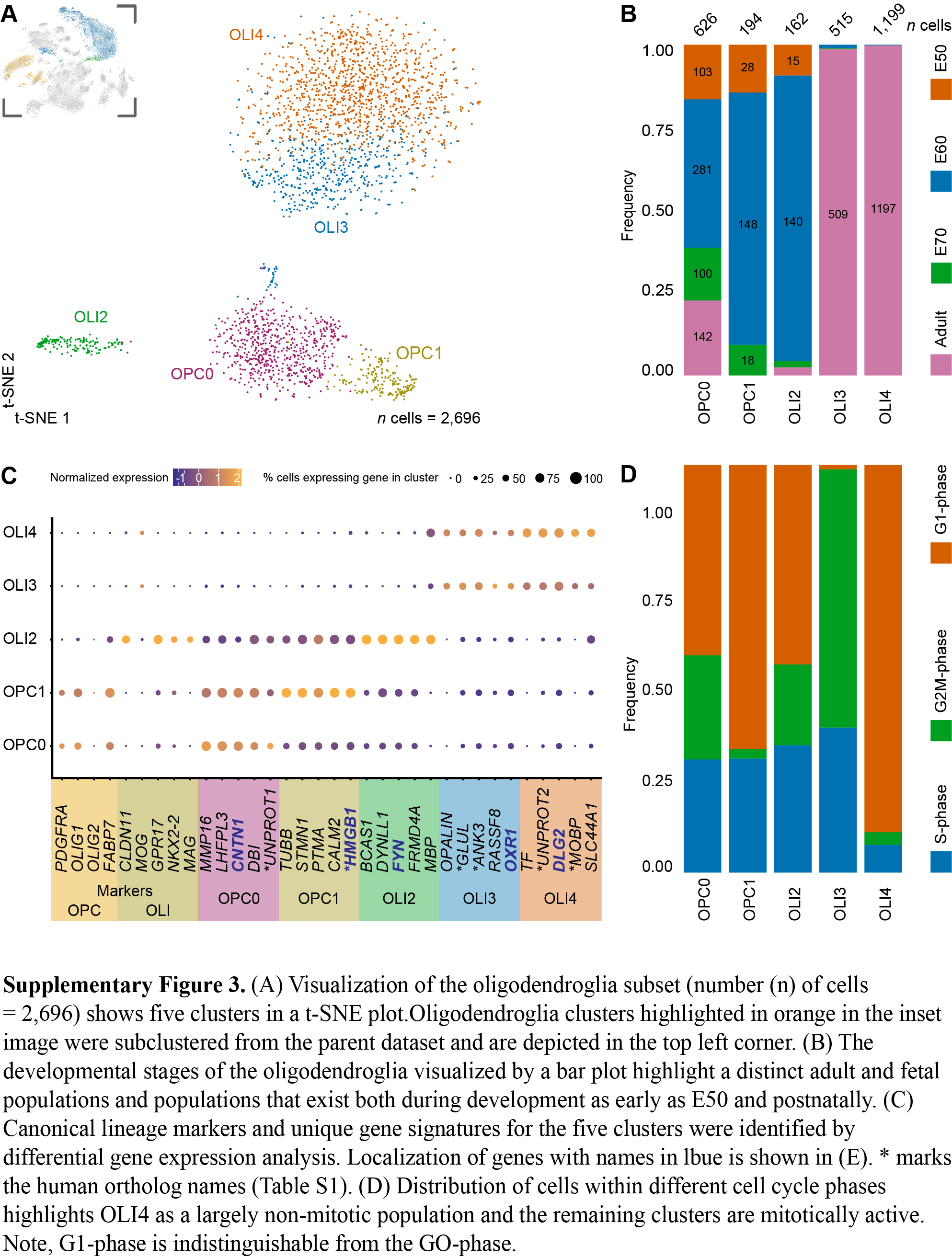

Supplement: Supplementary file 2 [file Image3.JPEG]

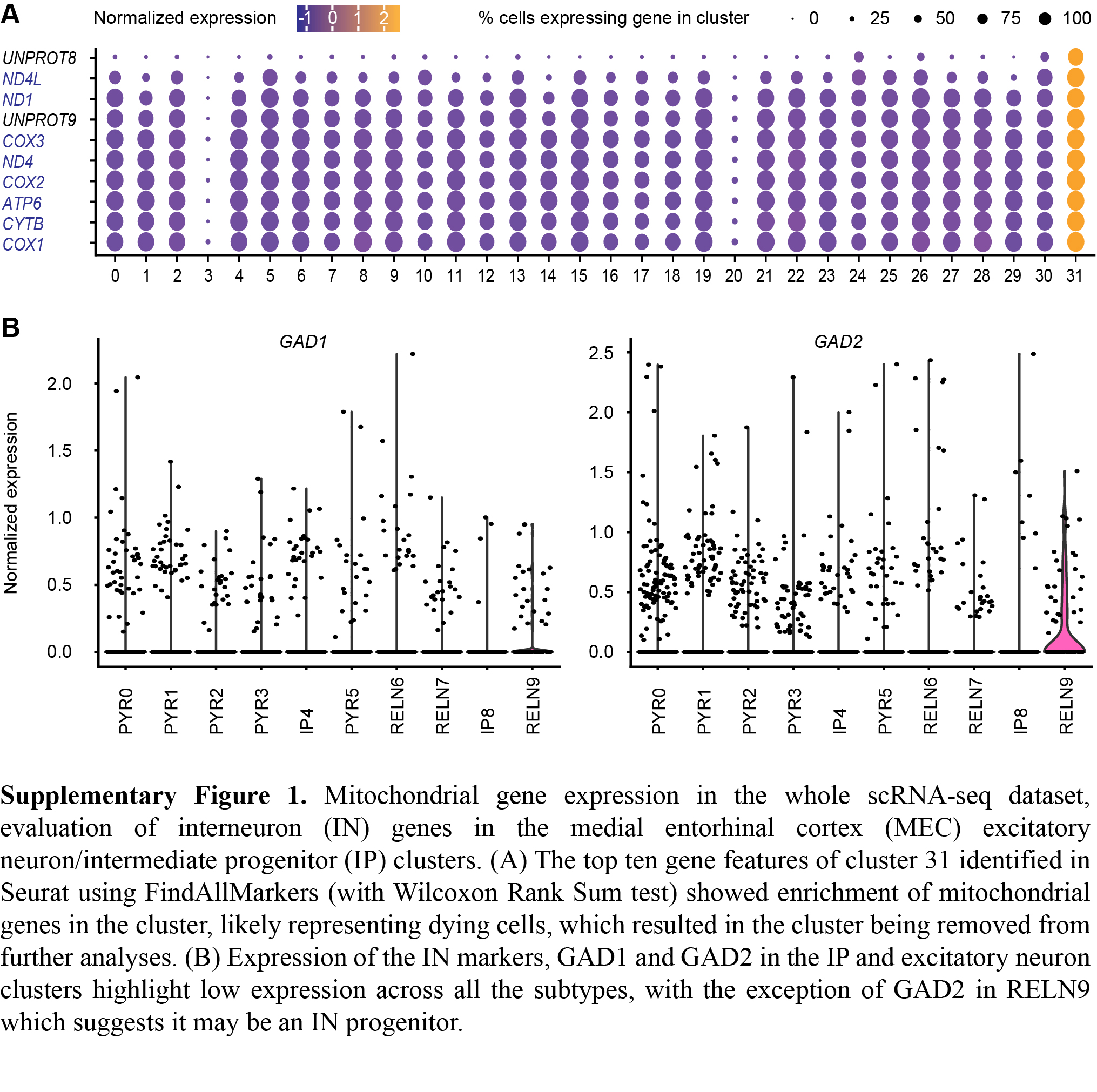

Supplement: Supplementary file 4 [file Image1.JPEG]

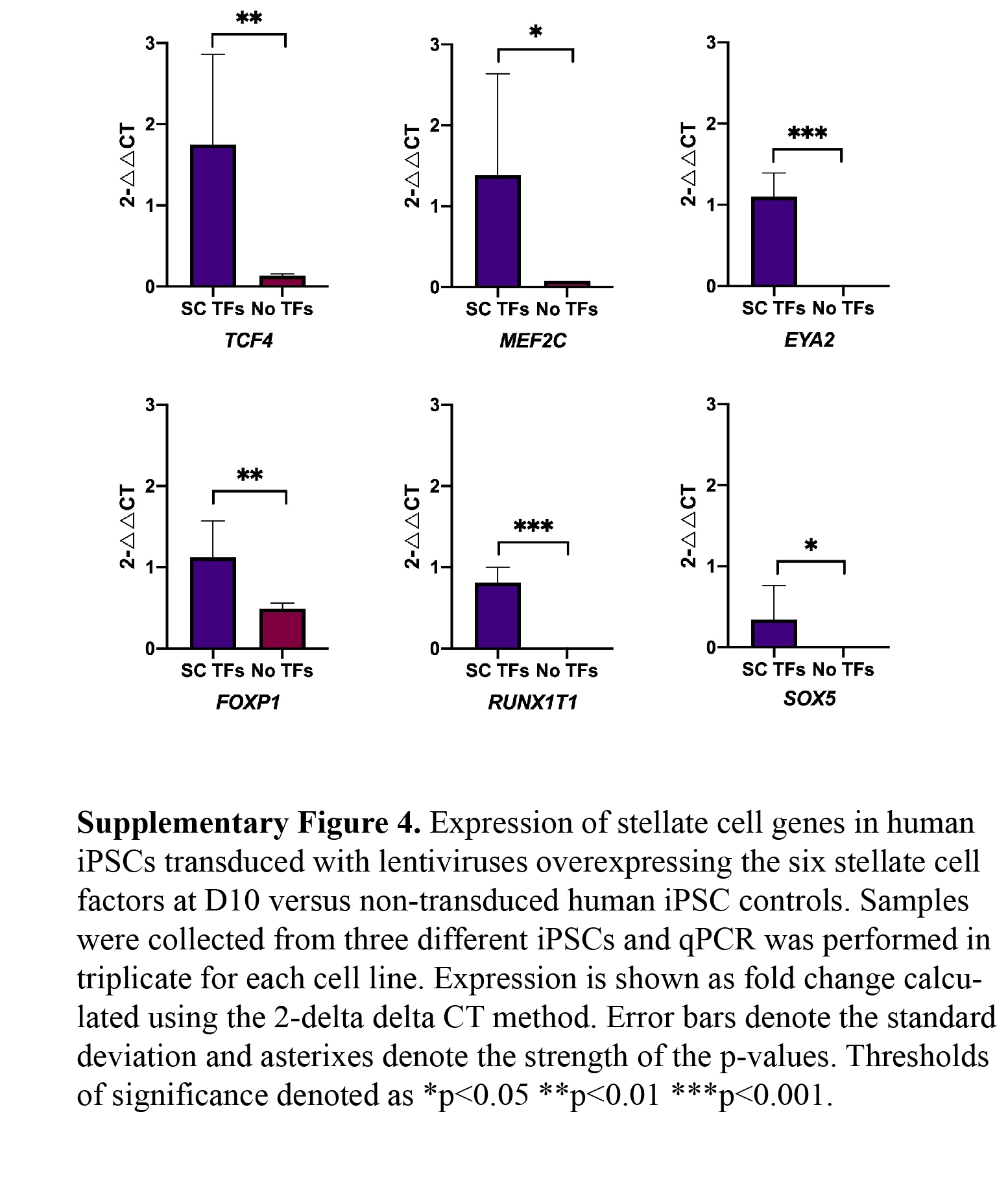

Supplement: Supplementary file 5 [file Image4.JPEG]

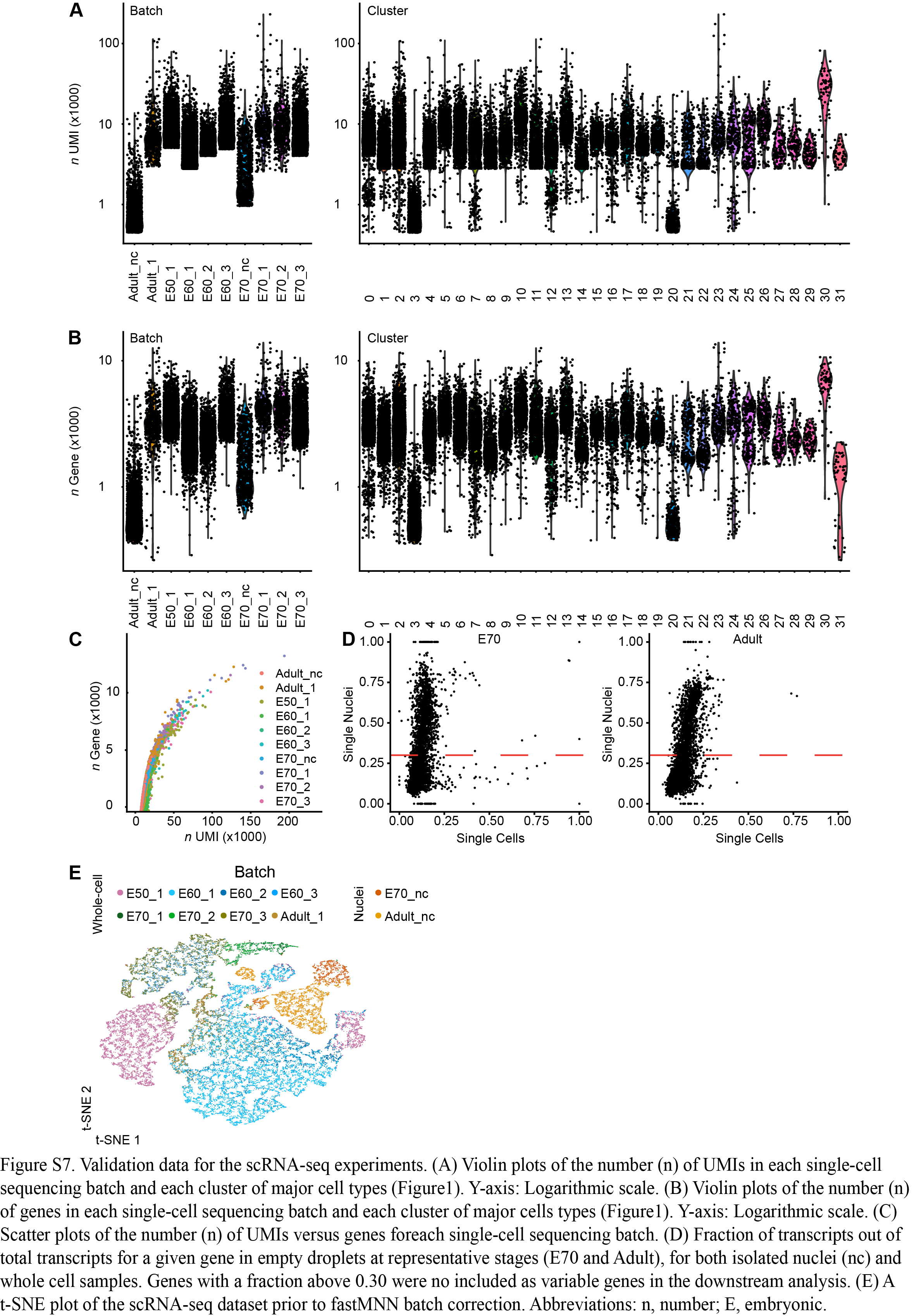

Supplement: Supplementary file 6 [file Image7.JPEG]

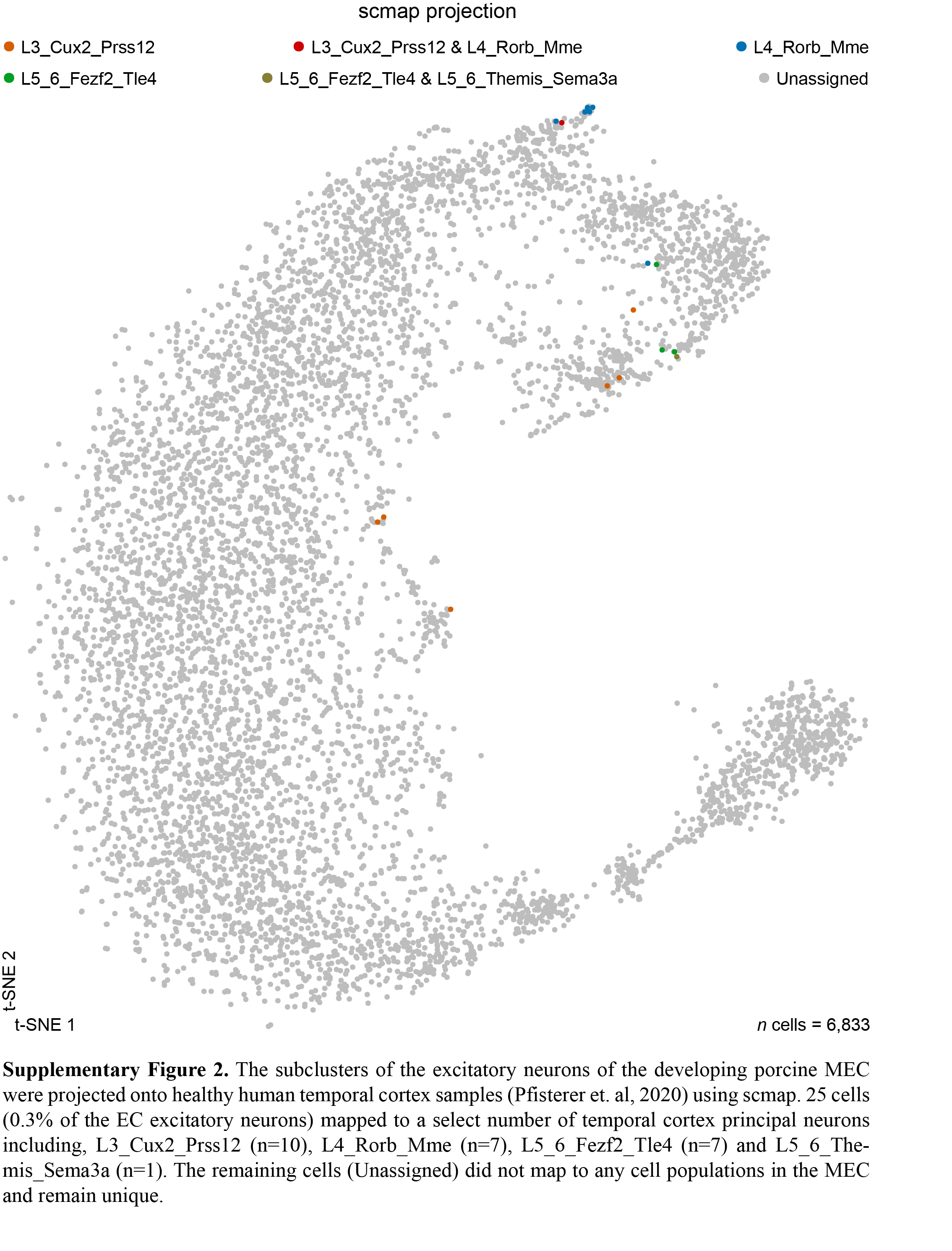

Supplement: Supplementary file 7 [file Image2.JPEG]

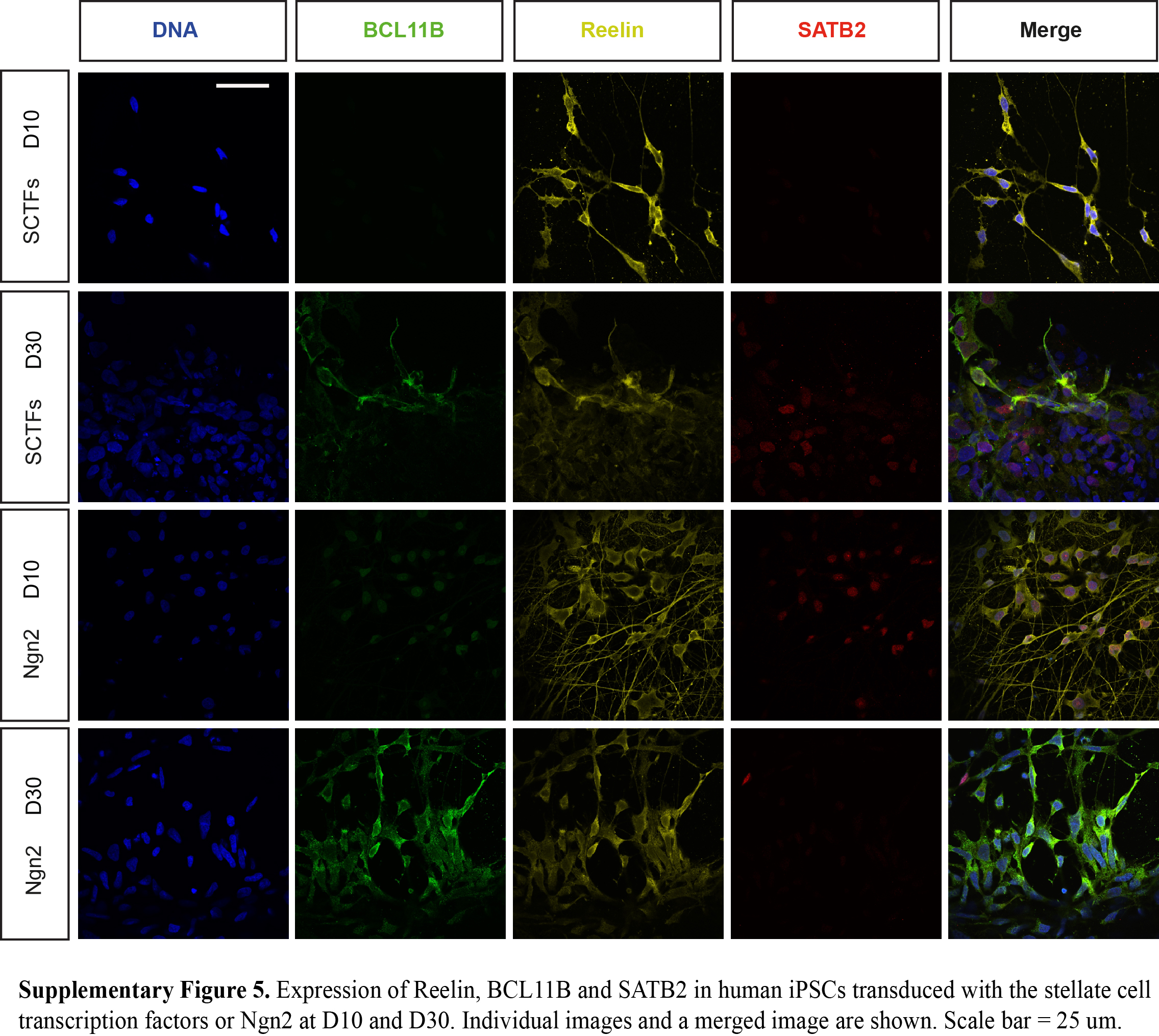

Supplement: Supplementary file 8 [file Image5.JPEG]

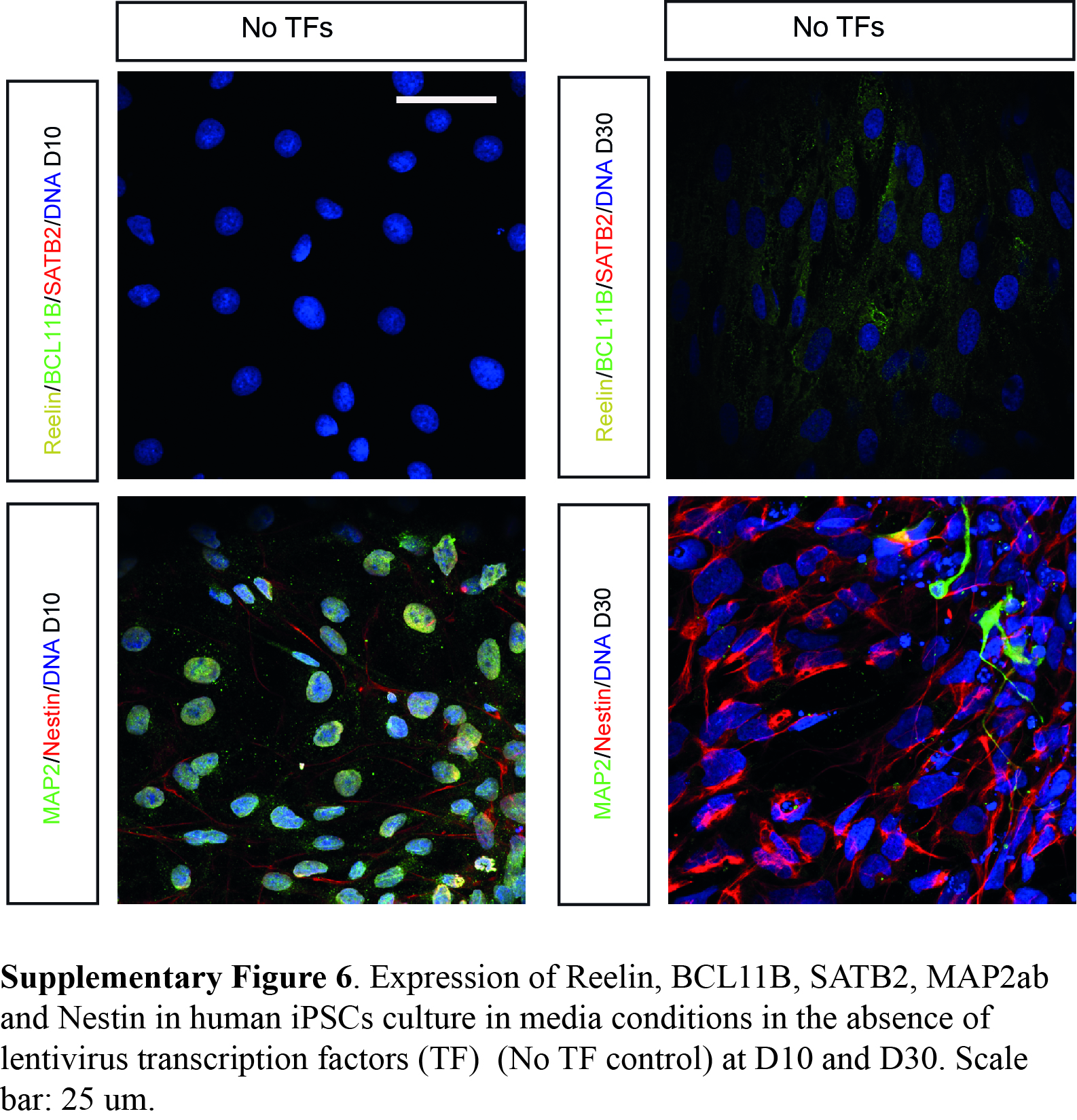

Supplement: Supplementary file 11 [file Image6.JPEG]
